# Supplementary material for: Optimization of fluorinated phenyl azides as universal photocrosslinkers for semiconducting polymers
Source: Nat Commun. 2024 Jul 28;15:6354. doi: 10.1038/s41467-024-50257-5 (PMC11284223; doi:10.1038/s41467-024-50257-5)

Supplementary Information: Optimization of fluorinated phenyl azides as universal photocrosslinkers for semiconducting polymers

Zhao-Siu Tan,[1#] Zaini Jamal,[1#] Desmond W. Y. Teo,[1] Hor-Cheng Ko[1], Zong-Long Seah,[2] Hao-Yu Phua[2], Peter K.H. Ho,[2] Rui-Qi Png,[2,*] Lay-Lay Chua[1,*]

[1] Department of Chemistry, National University of Singapore, Lower Kent Ridge Road, S117552, Singapore

[2] Department of Physics, National University of Singapore, Lower Kent Ridge Road, S117550, Singapore

[#] equal contribution

* phypngrq@nus.edu.sg; chmcll@nus.edu.sg

Supplementary Figures

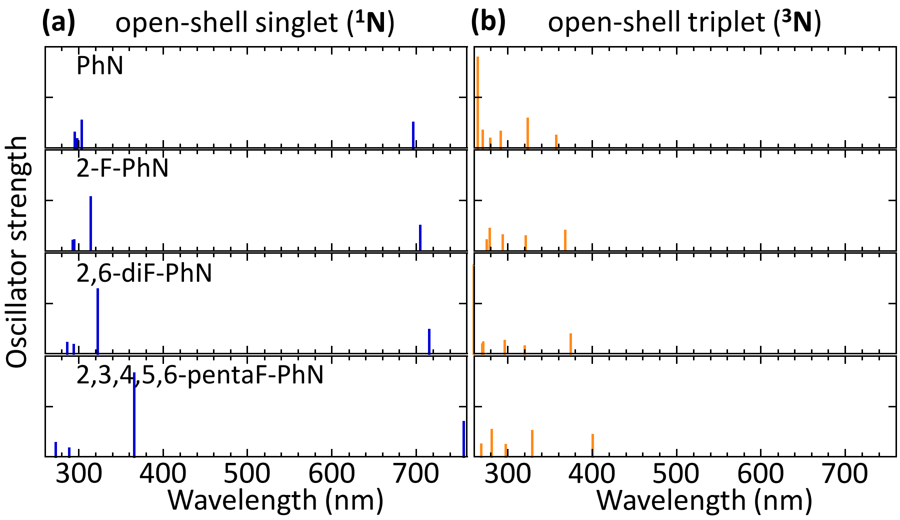

Supplementary Figure 1. Theoretical electronic transition spectra of selected phenyl and fluorinated phenylnitrenes. (a) Open-shell singlet nitrenes, **(b)** open-shell triplet nitrenes for: phenylnitrene; 2-fluorophenylnitrene; 2,6-difluorophenylnitrene; and 2,3,4,5,6-pentafluorophenylnitrene, computed with unrestricted Kohn-Sham TD-DFT/CAM-B3LYP/6-31G in acetonitrile SMD. Oscillator strength is 0.05 at full scale.

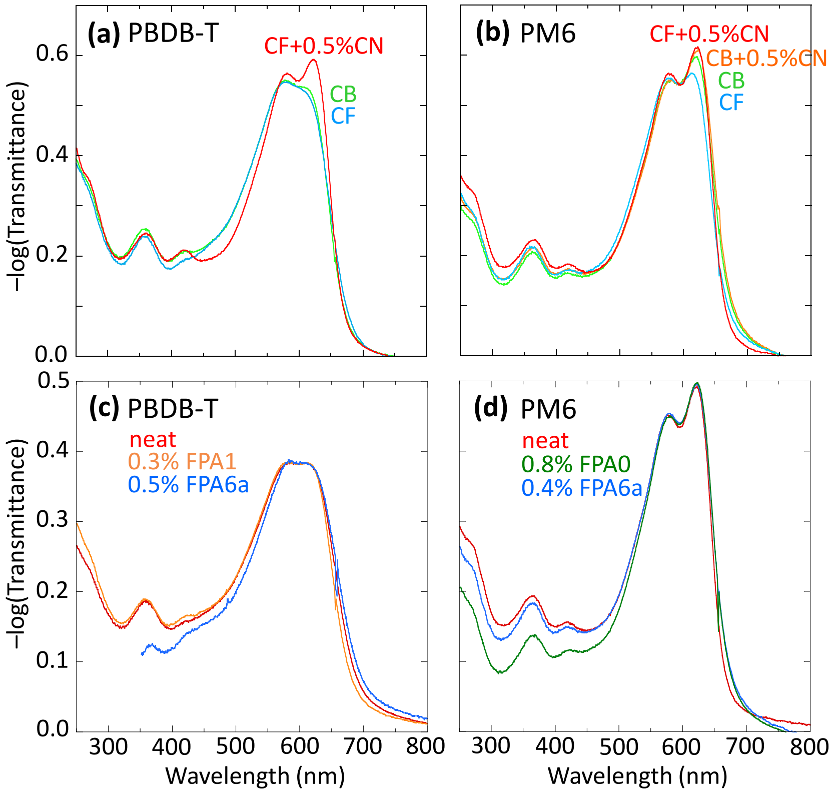

Supplementary Figure 2. UV–Vis–NIR spectroscopy of polymer films spin-cast from different solvents at room temperature. **(a)** PBDB-T and **(b)** PM6. Solvents: CB, chlorobenzene; CF, chloroform; CN, 1-chloronaphthalene; % given as volume ratio to main solvent. Solution concentration, 11 mg mL^{-1}. Film thickness, scaled to 85 nm for presentation. **(c)** PBDB-T from CB, and **(d)** PM6 from CF, without and with selected FPA at given w/w%. Film thickness, scaled to 50 and 80 nm for PBDB-T and PM6, respectively, for presentation.

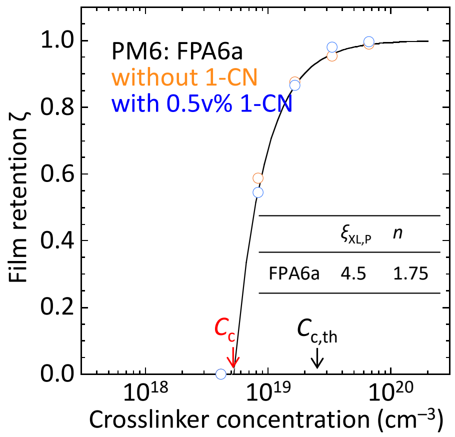

Supplementary Figure 3. Film retention characteristics for PM6: FPA6a, cast from chloroform, without and with 0.5 v% 1-chloronaphthalene as solvent modifier. Polymer characteristics: M_n 30 kD, $Đ$ 3.0. Data are fitted to: $\zeta = 1 - (C_c/C)^n$ as guide-to-the-eye, following Figure 5. The theoretical gel point ($C_{c,th}$) for this sample is 2.4×10^{19} cm^{-3}.

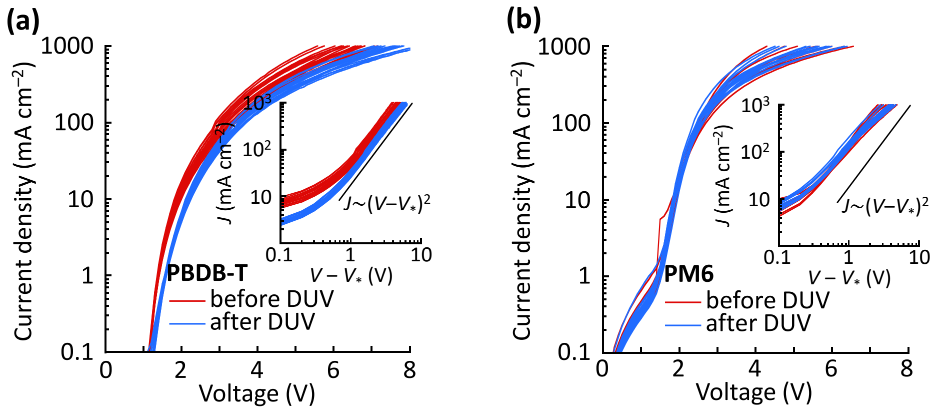

Supplementary Figure 4. Hole-only diodes to evaluate effect of 254-nm DUV on hole-carrier mobility: glass/ ITO/ PEDT: PSSH/ polymer/A g. (a) PBDB-T (red, pristine; blue, after DUV) and **(b)** PM6 (red, pristine; blue, after DUV). Spin-on PEDT:PSSH provides ohmic hole injection contact, evaporated Ag provides hole exit contact. Films were photo-exposed at 254-nm wavelength from a low-pressure Hg lamp (dose, 200 mJ cm^{-2}) in a nitrogen glovebox before Ag deposition. Second sweep data in the high-to-low direction are shown for representative diodes. Film thicknesses, 100 nm (for PBDB-T), 75 nm (PM6). Insets: Mott–Gurney plots.

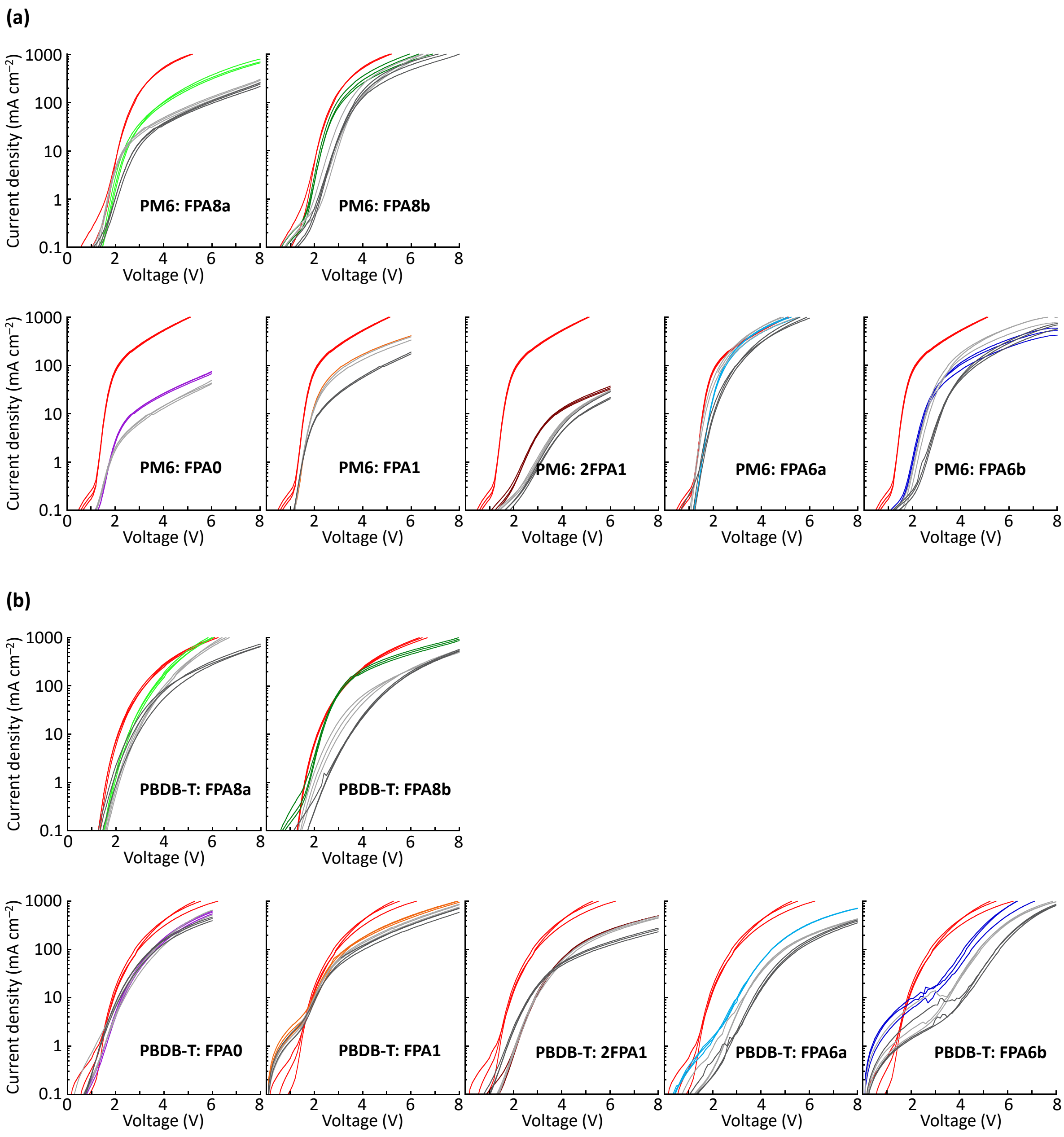

Supplementary Figure 5. Hole-only diodes to measure effect of FPA photocrosslinking on hole-carrier mobility: glass/ ITO/ PEDT: PSSH/ polymer: FPA/ Ag. (a) PM6: FPA, and **(b)** PBDB-T: FPA. FPA concentration: red, no FPA; other colour, 0.5 w/w%; grey, 1 w/w%; black, 2 w/w%. Spin-on PEDT:PSSH provides ohmic hole injection contact, evaporated Ag provides hole exit contact. Films were photo-exposed at 254-nm wavelength (200 mJ cm^{-2}) in a nitrogen glovebox before Ag deposition. Second sweep data in the high-to-low direction are shown for representative diodes. Film thicknesses, 95 nm (for set of PM6: FPA8 and PM6: 8b) or 85 nm (others). The pristine devices (i.e. no FPA) are identical in each set.

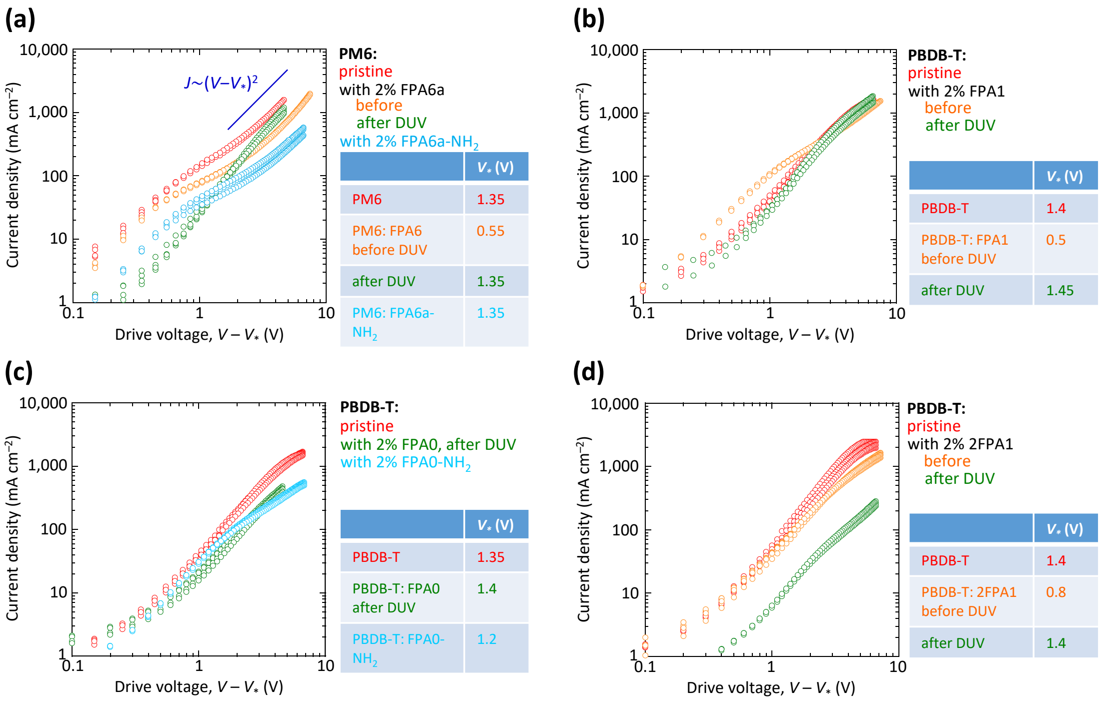

Supplementary Figure 6. Hole-only diodes to probe chemical and photochemical effects: glass/ ITO/ PEDT: PSSH/ polymer: FPA/ Ag. (a) PM6, **(b)–(d)** PBDB-T. The apparent built-in potential (V_*) is obtained by fitting to the Mott–Gurney equation: $J = \frac{9}{8}\varepsilon\mu\frac{(V-V_*)^2}{d^3}$, and compiled in accompanying tables. Three representative diodes shown for each case. Adding FPA downshifts V_* by 0.6–0.9 V, but DUV exposure recovers the original value upon azide photolysis.

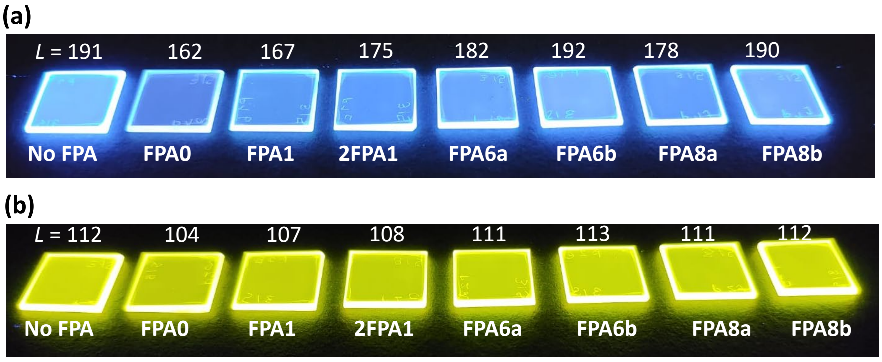

Supplementary Figure 7. Photoluminescence image of (a) blue and (b) yellow light-emitting polymer semiconductors, excited at 254-nm wavelength. Films containing 0.5 w/w % of FPA crosslinkers were exposed to 254-nm wavelength (dose, 200 mJ cm^{-2}) in nitrogen glovebox, and imaged at 254-nm excitation. *L* gives the lightness value in the hue–saturation–lightness (HSL) colour model for the fluorescence emitted through the front face. No fluorescence quenching is observed with FPA6b and 8b for the blue polymer, and with FPA6a, 6b, 8a and 8b for the yellow polymer.

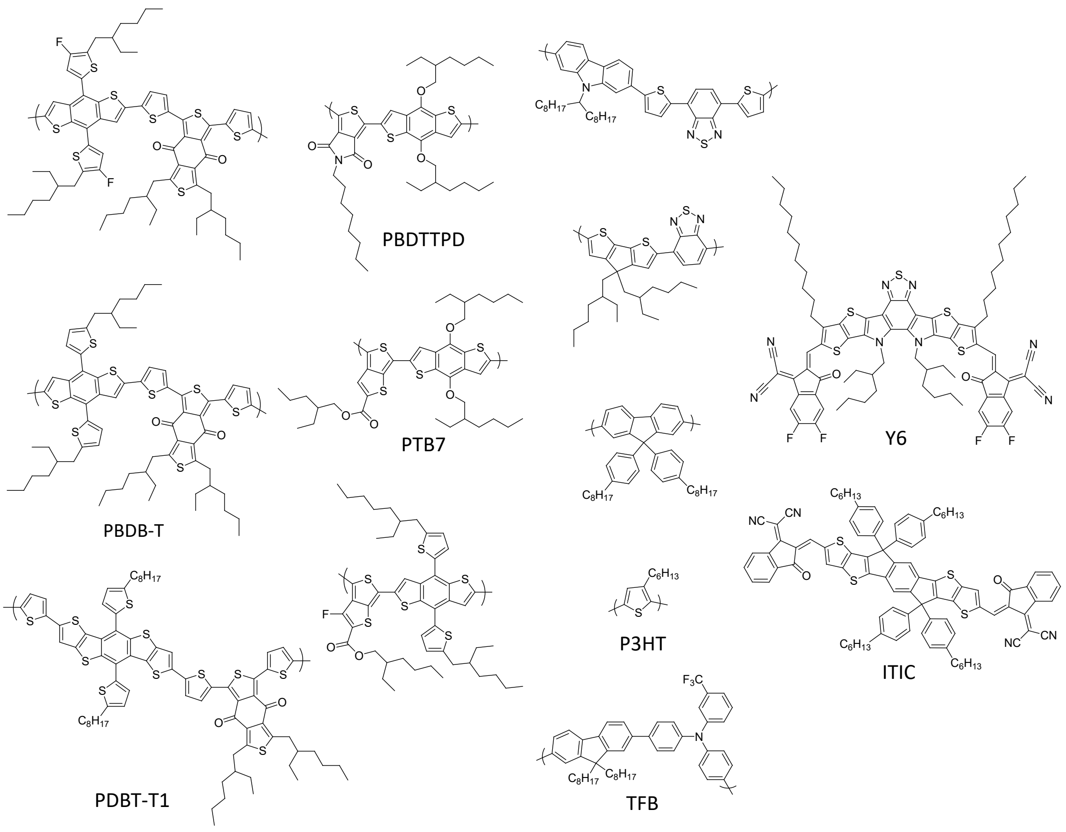

Supplementary Figure 8. Chemical structure of organic semiconductors in Figure 10. PM6, PBDB-T, PDBT-T1, PBDTTPD, PCDTBT, PCPDTBT, PTB7 and PTB7-Th are polymer donors. Y6 and ITIC are non-fullerene acceptors. PFOP, P3HT and TFB are polymer semiconductors.

Supplementary Tables

Crosslinker	Melting temperature (T_m/°C)[a]	Onset loss temperature (T_{loss}/°C)[b]	Weight loss (%)[c]	Decomposition enthalpy (Q_{dec}/J g^{-1})[d]	Shock sensitivity index (S)[e]	Explosive propagation index (E)[e]
**FPA0**	133	119	22.7 (13.7)	1,210	–0.01	0.01
**FPA1**	78	119	14.1 (11.3)	1,010	–0.11	–0.08
**2FPA1**	138	128	11.1 (11.2)	1,050	–0.09	–0.07
**FPA8a**	72	133	12.6 (11.5)	990	–0.13	–0.10
**FPA8b**	135	131	13.8 (11.8)	1,010	–0.12	–0.09
**FPA6a**	--	131	11.1 (10.3)	920	–0.15	–0.13
**FPA6b**	106	132	10.3 (9.5)	710	–0.27	–0.24
**PA1**	89	150	18.3 (15.9)	1,300	–0.05	0.00

Footnotes:

[a] evaluated for dominant polymorph from differential scanning calorimetry in hermetically-sealed Al pans in nitrogen

[b] evaluated as temperature at which the combined rate of azide decomposition and any sublimation first exceeds 1% min^{-1}, i.e., sample loses 5% of azide after 5 min

[c] evaluated from thermogravimetry, theoretical value for N_2 loss given in brackets; FPA0, FPA1 and PA1 exhibit significant additional losses due to sublimation, e.g. FPA0, 22.7% (expt) *vs* 13.7% (theo).

[d] evaluated from exotherm integration, differential scanning calorimetry

[e] evaluated from Yoshida correlations: $S = \log(Q_{dec}/4.18) - 0.72 * \log(T_{dec} - 25) - 0.98$; $E = \log(Q_{dec}/4.18) - 0.38 * \log(T_{dec} - 25) - 1.67$, where T_{dec} is the extrapolated decomposition onset temperature (in °C), and Q_{dec} given in J g^{-1}; compound is unlikely to be explosive if both S and E are negative

Supplementary Table 1. Thermophysical properties of FPA photocrosslinkers. PA1 is the unfluorinated analogue of FPA1 for comparison.

Crosslinker	Molar absorptivity (ε/ 10^4 M^{-1} cm^{-1})[a]	Photo-absorption cross-section (σ/ 10^{-16} cm^2)[b]	Irradiance (E_e/ mW cm^{-2})	Film thickness (nm)	Photolysis time constant (t_o/ s)[c]	Photolysis quantum efficiency (φ_P)[d]	Crosslinking efficiency ($\xi_{XL,P}$)[e]
**FPA0**	1.27	0.49	0.93	400	28.5	1.0	0.6
**FPA1**	2.58	0.99	0.74	500	19.4	1.0	0.95
**2FPA1**	4.85	1.85	0.74	650	21.6	1.0	0.95
**FPA8a**	2.84	1.09	1.10	250	11.7	0.95	0.95
**FPA8b**	2.56	0.98	1.10	250	11.7	0.95	0.7
**FPA6a**	3.09	1.18	0.74	600	16.2	1.0	1.05
**FPA6b**	2.84	1.09	0.74	450	16.2	1.05	0.45

Footnotes:

[a] Evaluated at 254-nm wavelength from absorption spectra in acetonitrile

[b] Computed from: $\sigma = \varepsilon \ln(10)\ (10^3\ cm^3\ L^{-1})\ /(6.022 \times 10^{23}\ mol^{-1})$

[c] Measured in PS film (M_n 200 kD; M_w 202 kD) by fitting the time-dependent intensity of azide vibration (ν_{as} N_3) mode to exponential decay against 254-nm radiant exposure. The irradiance (E_e) was measured using a standard SiC photodiode. An E_e of 1.10 mW cm^{-2} corresponds to a photon irradiance (E_q) of 1.41 x 10^{15} cm^{-2} s^{-1}. Concentration, 5–10 w/w% FPA in PS. Estimated uncertainty in t_o is 0.5 s for 1 standard deviation (sd).

[d] Evaluated for each azide group from: $\varphi_P = t_{o,th}\ t_o^{-1}$, where theoretical time constant $t_{o,th}$ is $(E_q\ \xi\ \sigma\ /2)^{-1}$ for bisazide and $(E_q\ \xi\ \sigma\ /4)^{-1}$ for tetrakisazide, where ξ is the absorptance enhancement factor due to thin-film interference, and the divisors 2 or 4 account for number of azide groups. Estimated uncertainty in φ_P is 0.05 (1 sd), dominated by uncertainty in irradiance. FPA1 and FPA6 were also re-tested at 1.10 mW cm^{-2} to confirm results, validating methodology.

[e] Evaluated for polystyrene films (200-nm-thick PS: FPA): $\xi_{XL,P} = C_{c,th}/C_c$, where $C_{c,th}$ is the theoretical crosslinker concentration to reach the gel point, given by $C_{c,th} = \frac{\rho_P N_A}{M_n}$ and $C'_{c,th} = \frac{1}{2}\frac{\rho_P N_A}{M_n}$ for bisazide and tetrakisazide, respectively, where M_n is the number average molar mass of the polymer, and C_c is the experimental concentration for the gel point, given by the take-off in the plot of film retention ratio against crosslinker concentration. Estimated uncertainty in $\xi_{XL,P}$ is 0.05 (1 sd).

Supplementary Table 2. Photophysical properties of FPA photocrosslinkers.

FPA unit[a]	ΔU_{xA} (eV)[b] Binding in amorphous alkane phase	ΔU_{xX} (eV)[c] Binding in amorphous FPA phase	ΔU_{xPS} (eV)[d] Binding in amorphous PS phase	ΔU_{xP} (eV)[e] Binding to polymer backbone	ΔU_{PP} (eV)[f] Polymer–polymer binding	ΔU_{PxP} (eV)[g] Binding in tight sandwich phase	$\Delta\Delta U_{A–X}$ (eV)[h] Binding in alkane phase rel to FPA phase	$\Delta\Delta U_{PS–X}$ (eV)[i] Binding in PS phase rel to FPA phase	$\Delta\Delta U_{P–A}$ (eV)[j] Binding in loose sandwich rel to side-chain phase	$\Delta\Delta U_{PP–A}$ (eV)[k] Binding in tight sandwich rel to side-chain phase
**FPA0**	–1.14	–1.12	–1.13	–0.91	–1.10	–0.72	-0.02	-0.01	0.23	0.42
**Hemi-FPA1**	–0.91	–0.69	–0.70	–0.76	–1.10	–0.42	-0.22	-0.01	0.15	0.49
**Hemi-FPA8a**	–1.11	–0.65	–0.79	–0.83	–1.10	–0.56	-0.46	-0.14	0.28	0.55
**Hemi-FPA8b**	–1.06	–0.83	–0.70	–0.83	–1.20	–0.46	-0.23	0.13	0.23	0.60
**Hemi-FPA6a**	–0.99	–0.61	–0.68	–0.86	–1.35	–0.37	-0.38	-0.07	0.13	0.62
**Hemi-FPA6b**	–1.06	–0.79	–0.78	–0.93	–1.35	–0.51	-0.27	0.01	0.13	0.55
**Hemi-PA1**	–1.07	–0.75	–0.58	–0.78	–1.10	–0.46	-0.32	0.17	0.29	0.61

Footnotes:

[a] FPA unit refers to FPA molecule for FPA0, and hemi-FPA (i.e. half crosslinker) for the others, where each half is capable of separate interactions with the local nanophases.

[b] Binding energy of FPA unit in amorphous alkane model cluster: alkane cluster + FPA → FPA embedded in alkane cluster, where the alkane model cluster comprises sixteen 5,12-diethylhexadecane molecules. Computed standard error of mean (σ) is 0.09 eV.

[c] Binding energy of FPA unit in amorphous FPA cluster: n FPA molecule + FPA molecule → (n+1) FPA molecule, where the FPA cluster comprises fifteen FPA molecules. The results are normalized to unit size (σ = 0.1 eV). The amorphous FPA cluster is generated by heating to 450 K then quenching to 200 K.

[d] Binding energy of FPA unit in amorphous polystyrene cluster: PS cluster + FPA → FPA embedded in PS cluster, where the PS cluster comprises sixteen PS pentamers. σ = 0.09 eV.

[e] Binding energy of FPA unit in polymer backbone/FPA complex: polymer + FPA → polymer/FPA complex, where the polymer backbone is PM6 or PBDB-T. σ = 0.035 eV.

[f] Polymer–polymer backbone binding energy over the disruption length of FPA unit: polymer + polymer → polymer/polymer π-stacked double-chain, where the polymer backbone is PM6 or PBDB-T. σ = 0.035 eV.

[g] Binding energy of FPA unit in polymer/FPA/polymer tight sandwich: polymer/polymer π-stacked double-chain + FPA → polymer/FPA/polymer tight sandwich, computed as $\Delta U_{PxP} = 2*\Delta U_{xP} – \Delta U_{PP}$. Estimated σ = 0.1 eV.

[h] Change in binding energy for FPA in alkane phase relative to FPA phase, computed as $\Delta\Delta U_{A–X} = \Delta U_{xA} – \Delta U_{xX}$. Estimated σ = 0.1 eV.

[i] Change in binding energy for FPA in PS phase relative to FPA phase, computed as $\Delta\Delta U_{PS–X} = \Delta U_{xPS} – \Delta U_{xX}$. Estimated σ = 0.1 eV.

[j] Change in binding energy for FPA in loose-sandwich backbone phase relative to alkane phase, computed as $\Delta\Delta U_{P–A} = \Delta U_{xP} – \Delta U_{xA}$. Estimated σ = 0.1 eV.

[k] Change in binding energy for FPA in tight-sandwich backbone phase relative to alkane phase, computed as $\Delta\Delta U_{PP–A} = \Delta U_{PxP} – \Delta U_{xA}$. Estimated σ = 0.1 eV.

Supplementary Table 3. Local mean interaction energies of FPA crosslinkers computed by OPLS4 molecular forcefield.

S/N	Sample[a]	Theo atom ratio (S : F : C)	Exptal atom ratio (S : F : C)	Surface enrichment[b]
1a	PBDB-T	1 : 0.000 : 8.62	1 : 0.000 : 8.55	--
1b	PBDB-T: FPA0	1 : 0.059 : 8.72	1 : 0.12 : 8.5	2.0
1c	PBDB-T: FPA1	1 : 0.048 : 8.66	1 : 0.088 : 8.65	1.8
1d	PBDB-T: 2FPA1	1 : 0.048 : 8.64	1 : 0.053 : 8.7	1.4
1e	PBDB-T: FPA6a	1 : 0.033 : 8.66	1 : 0.040 : 8.7	1.2
1f	PBDB-T: FPA6b	1 : 0.020 : 8.66	1 : 0.025 : 8.65	1.3
2a	PM6	1 : 0.250 : 8.62	1 : 0.33 : 8.4	--
2b	PM6: FPA0	1 : 0.310 : 8.72	1 : 0.42 : 8.8	1.5
2c	PM6: FPA1	1 : 0.298 : 8.72	1 : 0.49 : 8.75	3.2
2d	PM6: 2FPA1	1 : 0.298 : 8.72	1 : 0.39 : 8.5	1.6
2e	PM6: FPA6a	1 : 0.283 : 8.75	1 : 0.38 : 8.5	1.7
2f	PM6: FPA6b	1 : 0.270 : 8.77	1 : 0.37 : 8.4	2.0

Footnotes:
[a] FPA crosslinker is added at 2.0 w/w%.
[b] Surface enrichment factor. For PBDB-T: FPA films, this is given by ratio of experimental F/S stoichiometry $(F/S)_{ex}$ to theoretical F/S stoichiometry $(F/S)_{th}$; for PM6: FPA films, ratio of experimental $[(F/S)_{ex} – (F/S)_{ex,PM6}]$ to theoretical $[(F/S)_{th} – (F/S)_{th,PM6}]$, due to presence of F in polymer matrix. For 2FPA1 crosslinker, half of the $(F/S)_{th}$ ratio is scaled by 60% to account for lower visibility of the lower half of the molecule located deeper from film surface, based on inelastic mean free path consideration. All sulfur atoms arise only from the polymer.

Supplementary Table 4. Theoretical and experimental atomic ratio by XPS core-level spectroscopy of PM6 and PBDB-T. Photoelectron take-off angle, 90°.

Supplementary Methods

Synthesis of FPA1

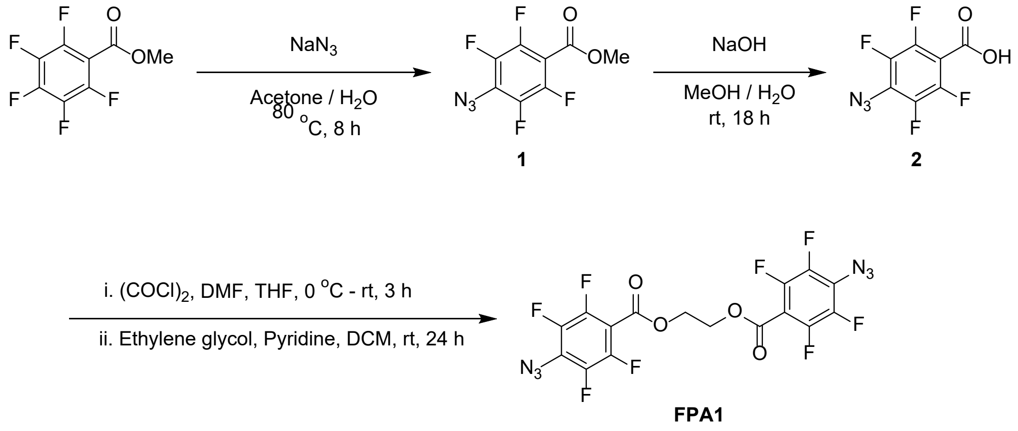

Methyl 4-azido-2,3,5,6-tetrafluorobenzoate (1): To a 2-neck 100-mL RBF equipped with a magnetic stir bar and a reflux condenser, methyl 2,3,4,5,6-pentafluorobenzoate (4.4 mL, 30 mmol), 50 mL acetone, 20 mL water and NaN_3 (2.34 g, 36 mmol) were mixed. After 8 h of reflux at 80 °C, the reaction mixture was cooled down to room temperature and acetone removed under reduced pressure followed by extraction of the remaining aqueous suspension with diethyl ether (20 mL x 3). The combined organic layer was then dried over Na_2SO_4, filtered and concentrated to dryness under reduced pressure to afford **1** as white solid in quantitative yield (7.48 g). ^{1}H NMR (400 MHz, $CDCl_3$): δ 3.96 (s, 3H); ^{19}F NMR (377 MHz, $CDCl_3$): δ -151.01 – -150.92 (m, 2F), -138.74 – -138.65 (m, 2F).

4-azido-2,3,5,6-tetrafluorobenzoic acid (2): To a 100-mL RBF equipped with a magnetic stir bar, **1** (7.47 g, 30 mmol) was dissolved in 90 mL MeOH and 9.0 mL water. To the resulting solution, 10.5 mL 20% NaOH was slowly added. After 18 h of stirring at room temperature, the reaction mixture was diluted with water and the resulting aqueous layer was washed with

dichloromethane to remove unreacted organics. The aqueous layer was then acidified with 2.0 M HCl and extracted with dichloromethane (100 mL x 3). The combined organic layer was washed with brine, dried over Na_2SO_4, filtered and concentrated to dryness under reduced pressure to afford **2** as white solid in 74% yield (5.25 g). ^1H NMR (400 MHz, $CDCl_3$): δ 10.21 (brs, 1H); ^{19}F NMR (377 MHz, $CDCl_3$): δ -150.66 – -150.56 (m, 2F), -136.81 – -136.71 (m, 2F).

Ethane-1,2-diyl bis(4-azido-2,3,5,6-tetrafluorobenzoate) (FPA1): To an oven-dried 3-neck 25-mL RBF equipped with a magnetic stir bar, **2** (1.03 g, 4.4 mmol) was dissolved in 15 mL anhydrous THF under an argon atmosphere. At 0 °C (ice-water bath), oxalyl chloride (1.1 mL, 13.0 mmol) and 3 drops of anhydrous DMF were then slowly added. The resulting mixture was then allowed to warm up to room temperature. After 3 h, the reaction mixture was directly concentrated to dryness under reduced pressure to afford the intermediate 4-azido-2,3,5,6-tetrafluorobenzoyl chloride. Under an argon atmosphere, ethylene glycol (0.11 mL, 2.0 mmol) was added to the same 3-neck 25-mL RBF containing the crude intermediate. The mixture was then dissolved in 15 mL anhydrous dichloromethane and cooled down to 0 °C followed by the dropwise addition of pyridine (0.68 mL, 8.4 mmol). After 24 h of stirring at room temperature, the reaction mixture was poured into water, extracted with dichloromethane, washed with brine, dried over Na_2SO_4, filtered and concentrated to dryness under reduced pressure. Purification by recrystallization from MeOH afforded **FPA1** as white solid in 50% yield (497.2 mg). ^1H NMR (400 MHz, $CDCl_3$): δ 4.68 (s, 4H); ^{19}F NMR (377 MHz, $CDCl_3$): δ -150.77 – -150.67 (m, 4F), -138.23 – -138.14 (m, 4F).

Synthesis of 2FPA1

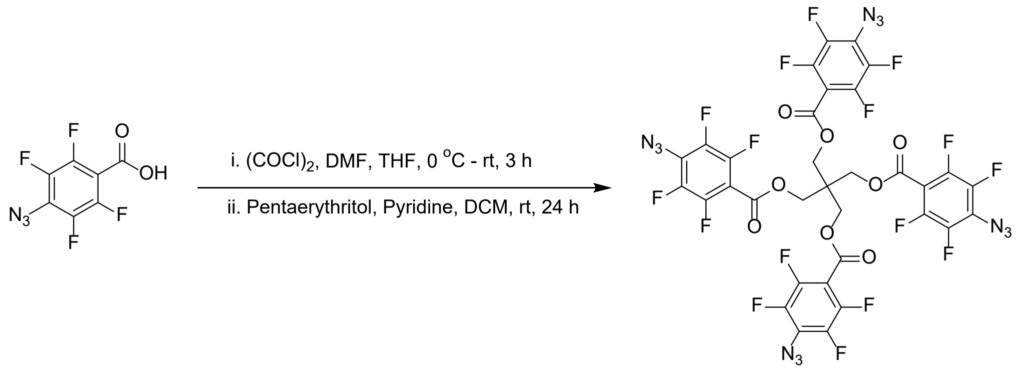

2FPA1

2,2-bis(((4-azido-2,3,5,6-tetrafluorobenzoyl)oxy)methyl)propane-1,3-diyl bis(4-azido-2,3,5,6-tetrafluorobenzoate) (2FPA1): To an oven-dried 3-neck 50-mL RBF equipped with a magnetic stir bar, **2** (1.98 g, 8.4 mmol) was dissolved in 25 mL anhydrous THF under an argon atmosphere and cooled to 0 °C (ice-water bath). Oxalyl chloride (2.2 mL, 25.2 mmol) and 3 drops of anhydrous DMF were slowly added. The resulting mixture was then allowed to warm up to room temperature. After 3 h, the reaction mixture was directly concentrated to dryness under reduced pressure to afford the intermediate 4-azido-2,3,5,6-tetrafluorobenzoyl chloride. Under an argon atmosphere, pentaerythritol (272.30 mg, 2.0 mmol) was added to the same 3-neck 50-mL RBF containing the crude intermediate. The mixture was then dissolved in 25 mL anhydrous dichloromethane and cooled down to 0 °C in ice-water bath followed by the dropwise addition of pyridine (1.4 mL, 16.8 mmol). After 24 h of stirring at room temperature, the reaction mixture was poured into water, extracted with dichloromethane, washed with brine, dried over Na_2SO_4, filtered and concentrated to dryness under reduced pressure. Purification by recrystallization from MeOH afforded 2**FPA1** as white solid in 73% yield (1.46 g). ^1H NMR (400 MHz, $CDCl_3$): δ 4.56 (s, 8H); ^{19}F NMR (377 MHz, $CDCl_3$): δ -150.32 – -150.22 (m, 8F), -138.04 – -137.95 (m, 8F).

Synthesis of FPA6a

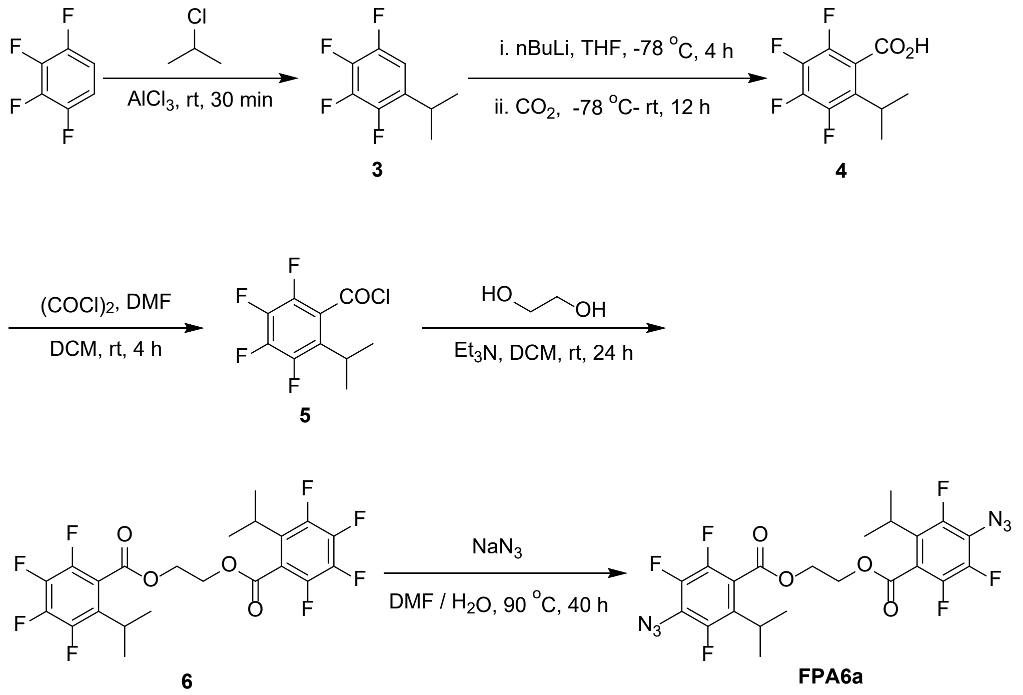

1,2,3,4-tetrafluoro-5-isopropylbenzene (3): To an oven-dried 100-mL RBF equipped with a magnetic stir bar, $AlCl_3$ (1.43 g, 10.8 mmol), 1,2,3,4-tetrafluorobenzene (8.1 g, 53.9 mmol) and 2-chloropropane (6.35 g, 80.8 mmol) were sequentially loaded. After 30 min of stirring at room temperature, water was then added and stirring was continued for another 10 min. The reaction mixture was extracted with diethyl ether (50 mL x 3) and the combined organic layer was washed with water and brine, dried over Na_2SO_4 and concentrated to dryness under reduced pressure. Crude **3** was obtained as a yellow oil in 53% yield (5.5 g) and used in the next step without further purification. ^{1}H NMR (300 MHz, $CDCl_3$): δ 6.82 (m, 1H), 3.22 (Sept, J = 6.75 Hz, 1H), 1.23 (d, J = 6.9 Hz, 6H); ^{19}F NMR (377 MHz, $CDCl_3$) δ: -160.15 – -160.01 (m, 1F), -156.69 – -156.59 (m, 1F), -145.28 – -145.17 (m, 1F), -140.24 – -140.13 (m, 1F).

2,3,4,5-tetrafluoro-6-isopropylbenzoic acid (4): To an oven-dried 2-neck 100-mL RBF equipped with a magnetic stir bar, **3** (5.2 g, 27.06 mmol) was dissolved in 45 mL anhydrous THF under an argon atmosphere. The resulting solution was then cooled down to -78 °C (dry

ice-acetone bath) and 2.0 M n-butyllithium in cyclohexane (14.88 mL, 29.76 mmol) was added dropwise. After 4 h of stirring at -78 ^oC, CO_2 gas was bubbled through while allowing the reaction mixture to gradually warm up to room temperature. After 12 h, the reaction mixture was acidified with 5% HCl and extracted with dichloromethane (50 mL x 3). The combined organic layer was washed with brine, dried over Na_2SO_4 and concentrated to dryness under reduced pressure. Crude **4** was obtained as white solid in 30% yield (1.9 g) and used in the next step without further purification. ^1H NMR (300 MHz, $CDCl_3$): δ 3.22 (Sept, J = 7.05 Hz, 1H), 1.43 (d, J = 5.58 Hz, 6H).

2,3,4,5-tetrafluoro-6-isopropylbenzoyl chloride (5): To an oven-dried 2-neck 50-mL RBF equipped with a magnetic stir bar, **4** (1.3 g, 5.5 mmol) was dissolved in 20 mL anhydrous dichloromethane under an argon atmosphere. Oxalyl chloride (1.39 g, 11.00 mmol) and 3 drops of anhydrous DMF were then slowly added. After 4 h of stirring at room temperature, the reaction mixture was concentrated to dryness under reduced pressure to obtain 2,3,4,5-tetrafluoro-6-isopropylbenzoyl chloride as light yellow oil in 98% yield (1.37 g). The acid chloride intermediate **5** was used directly for the next step without further purification.

Ethane-1,2-diyl bis(2,3,4,5-tetrafluoro-6-isopropylbenzoate) (6): To an oven-dried 50-mL RBF equipped with a magnetic stir bar, the freshly prepared crude **5** was dissolved in 20 mL anhydrous dichloromethane under an argon atmosphere. A mixture of ethylene glycol (0.15 g, 2.47 mmol) and triethylamine (0.55 g, 5.49 mmol) in 5.0 mL anhydrous dichloromethane was then added dropwise. After 24 h of stirring at room temperature, the reaction mixture was poured into water and extracted with dichloromethane (50 mL x 3). The combined organic layer was washed with brine, dried over Na_2SO_4 and concentrated to dryness under reduced pressure. Purification by silica gel chromatography (30% dichloromethane in

hexanes) gave **6** as colourless oil in 71% yield (880 mg). ^1H NMR (300 MHz, $CDCl_3$): δ 4.66 (s, 4H), 3.00 (Sept, J = 6.99 Hz, 2H), 1.30 (d, J = 5.58 Hz, 12H). ^{19}F NMR (377 MHz, $CDCl_3$) δ -157.84 – -157.72 (m, 1F), -152.32 – -152.21 (m, 1F), -140.91 – -140.81 (m, 1F), -139.59 – -139.51 (m, 1F).

Ethane-1,2-diyl bis(4-azido-2,3,5-trifluoro-6-isopropylbenzoate) (FPA6a): To an oven-dried 100-mL RBF equipped with a magnetic stir bar, **6** (0.796 g, 1.59 mmol) was dissolved in 40 mL anhydrous DMF. A solution of NaN_3 (0.52 g, 7.98 mmol) in 7.0 mL water was then added and the resulting mixture was subsequently heated to 90 °C for 40 h. After cooling down to room temperature, the reaction mixture was poured into water and extracted with ethyl acetate (50 mL x 3). The combined organic layer was washed with brine, dried over Na_2SO_4 and concentrated to dryness under reduced pressure. Purification by silica gel chromatography (35% dichloromethane in hexanes) afforded **FPA6a** as white solid in 70% yield (610 mg). ^1H NMR (300 MHz, $CDCl_3$): δ 4.64 (s, 4H), 2.98 (Sept, J = 7.05 Hz, 2H), 1.29 (dd, J_1 = 6.7 Hz, J_2 = 2.8 Hz, 12H). ^{19}F NMR (377 MHz, $CDCl_3$) δ -147.64 – -147.57 (m, 2F), -141.98 – -141.89 (m, 2F), -127.76 (d, *J* = 12.4 Hz, 2F).

Synthesis of FPA6b

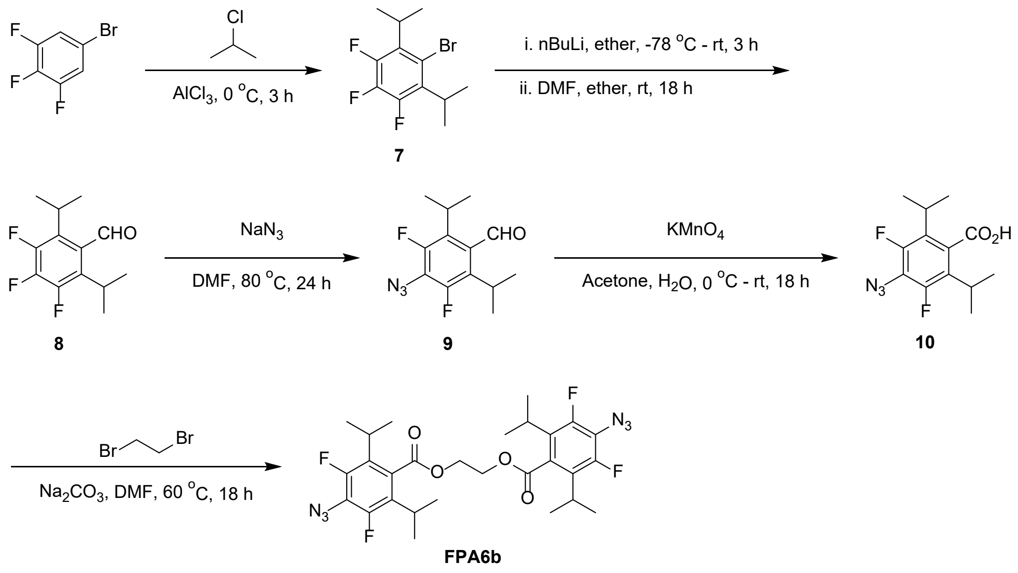

1-bromo-3,4,5-trifluoro-2,6-diisopropylbenzene (7): To an oven-dried 200-mL Schlenk tube equipped with a magnetic stir bar, $AlCl_3$ (6.70 g, 50 mmol) was loaded and sealed in N_2-filled glovebox. This schlenk tube was then submerged into 0 °C ice-water bath, followed by the dropwise addition of 5-bromo-1,2,3-trifluorobenzene (6.0 mL, 50 mmol) and 2-chloropropane (50 mL, 545 mmol) under positive pressure of argon with an outlet connected to a bubbler. The mixture was stirred for another 3 h at 0 °C. The resulting reaction mixture was then quenched with 50 mL 4.0 M NaOH, warmed up to room temperature, diluted with hexanes and passed through a short pad of silica gel. The hexanes layer was washed with water and brine followed by drying over Na_2SO_4 and then concentrated under reduced pressure. Purification by short-path vacuum distillation afforded **7** in 66% yield (9.68 g) as a colourless oil which solidified to white needle-like solid upon cooling in the freezer. ^{1}H NMR (400 MHz, $CDCl_3$): δ 1.33 (d, J = 7.1 Hz, 12H), 3.66 (sep, J = 7.1 Hz, 2H); ^{19}F NMR (377 MHz, $CDCl_3$): δ -160.82 (t, J = 20.2 Hz, 1F), -137.63 (d, J = 20.2 Hz, 2F).

3,4,5-trifluoro-2,6-diisopropylbenzaldehyde (8): To an oven-dried 100-mL two-neck RBF equipped with a magnetic stir bar, **7** (5.90 g, 20 mmol) was added. After 3-cycle of evacuation and purging with argon, 40 mL anhydrous diethylether was added to the RBF to dissolve the 1-bromo-3,4,5-trifluoro-2,6-diisopropylbenzene substrate. The reaction mixture was cooled to -78 ^oC (dry ice-acetone bath), then 2.0 M n-butyllithium in cyclohexane (11 mL, 22 mmol) was added dropwise. After 3 h of stirring at room temperature, anhydrous DMF (2.3 mL, 30 mmol) in 20 mL anhydrous diethyl ether was slowly added. The reaction mixture was left stirring at room temperature for 18 h then diluted with water. The organic layer was separated and further washed with brine followed by drying over Na_2SO_4 and concentrated under reduced pressure. The residue was purified by silica gel column chromatography (10% ethyl acetate in hexanes) to give **8** as a colourless oil in 63% yield (3.10 g). ^1H NMR (400 MHz, $CDCl_3$): δ 1.34 (d, *J* = 7.2 Hz, 12H), 3.28 (sep, *J* = 7.0 Hz, 2H), 10.48 (s, 1H); ^{19}F NMR (377 MHz, $CDCl_3$): δ -154.75 (t, *J* = 19.8 Hz, 1F), -137.07 (d, *J* = 20.0 Hz, 2F).

4-azido-3,5-difluoro-2,6-diisopropylbenzaldehyde (9): To an oven dried 100-mL RBF equipped with a magnetic stir bar, **8** (3.42 g, 14 mmol) and NaN_3 (1.82 g, 28 mmol) were added. Under an argon atmosphere, 40 mL anhydrous DMF was added and the mixture was then heated to 80°C in the dark for 24 h. Upon cooling to room temperature, the reaction mixture was diluted with water and extracted with dichloromethane (50 mL x 3). The combined organic layer was then washed with brine, dried over Na_2SO_4 and concentrated under reduced pressure. The residue was purified by silica gel column chromatography (5% ethyl acetate in hexanes) to give **9** as a light yellow oil in 97% yield (3.62 g). ^1H NMR (400 MHz, $CDCl_3$): δ 1.33 (d, *J* = 7.0 Hz, 12H), 3.29 (sep, *J* = 7.0 Hz, 2H), 10.47 (s, 1H); ^{19}F NMR (377 MHz, $CDCl_3$): δ -124.54 (s, 2F).

4-azido-3,5-difluoro-2,6-diisopropylbenzoic acid (10): To an oven dried 3-neck 250-mL RBF equipped with a magnetic stir bar and a 100-mL dropping funnel, **9** (2.68 g, 10 mmol) was dissolved in 60 mL acetone. At 0 ^{o}C (ice-water bath), a solution of $KMnO_4$ (1.90 g, 12 mmol) in 60 mL water was then slowly added through the dropping funnel. After 18 h of stirring at room temperature, the reaction mixture was diluted with water, washed with dichloromethane to remove unreacted organic and subsequently acidified with conc. HCl. The resulting aqueous layer was subsequently extracted with dichloromethane (100 mL x 3), washed with water and brine, dried over Na_2SO_4, filtered and concentrated to dryness under reduced pressure to afford **10** as off-white solid in 66% yield (1.87 g). ^{1}H NMR (400 MHz, DMSO-*d6*): δ 1.26 (d, *J* = 7.0 Hz, 12H), 2.86 (sep, *J* = 7.0 Hz, 2H), 13.72 (s, brs, 1H); ^{19}F NMR (377 MHz, DMSO-*d6*): δ -126.20

Ethane-1,2-diyl bis(4-azido-3,5-difluoro-2,6-diisopropylbenzoate) (FPA6b): To an oven dried 2-neck 50-mL RBF equipped with a magnetic stir bar, **10** (1.78 g, 6.3 mmol) and Na_2CO_3 (795 mg, 7.5 mmol) were mixed in 20 mL anhydrous DMF. After 5 min of stirring at room temperature, 1,2-dibromoethane (0.26 mL, 3.0 mmol) was slowly added and the resulting mixture was then heated to 60 ^{o}C. After 18 h, the reaction mixture was poured into water and extracted with dichloromethane (50 mL x 3). The combined organic layer was subsequently washed with water and brine, dried over Na_2SO_4, filtered and concentrated to dryness under reduced pressure. Purification by silica gel chromatography (30% dichloromethane in hexanes) afforded **FPA6b** as off-white solid in 82% yield (1.46 g). ^{1}H NMR (400 MHz, $CDCl_3$): δ 1.26 (d, *J* = 7.0 Hz, 24H), 2.70 (sep, *J* = 7.0 Hz, 4H), 4.58 (s, 4H); ^{19}F NMR (377 MHz, $CDCl_3$): δ -125.41 (s, 4F).

Synthesis of FPA0

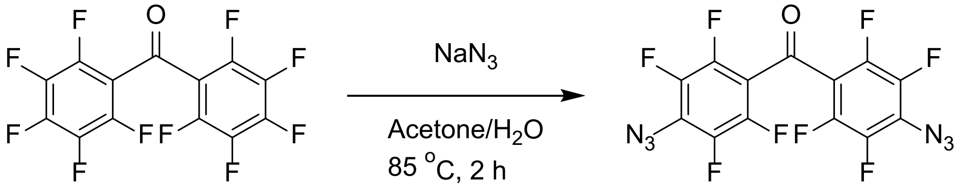

FPA0

Bis(4-azido-2,3,5,6-tetrafluorophenyl)methanone: To an oven dried two neck RBF equipped with a magnetic stir bar, bis(perfluorophenyl)methanone (1.0 g, 2.76 mmol) was dissolved in 20 mL acetone under an argon atmosphere. A solution of NaN_3 (0.37 g, 5.79 mmol) in 5.0 mL water was then slowly added. The reaction mixture was heated to 85 °C for 2 h. The reaction was then quenched by addition of ice cold water. The organic compound was extracted with ethyl acetate (50 mL x 3) and concentrated under reduced pressure. Purification by silica gel chromatography (5% ethyl acetate in hexane) gave **FPA0** as a white solid (1.07 g) in 95% yield. ^{19}F NMR ($CDCl_3$, 282 MHz) δ: -141.47 to -141.60 (m, 4F), -150.09 to -150.22 (m, 4F).

Synthesis of FPA0-NH$_2$

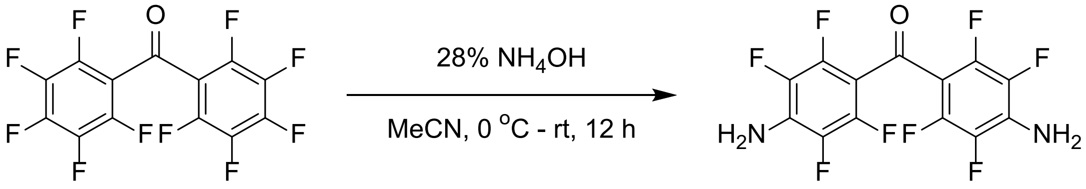

FPA0-NH$_2$

Bis(4-amino-2,3,5,6-tetrafluorophenyl)methanone (FPA0-NH$_2$): To a 25-mL RBF equipped with a magnetic stir bar, bis(perfluorophenyl)methanone (1.12 g, 3.1 mmol) was dissolved in 4.0 mL MeCN. To this solution at 0 °C (ice-water bath), 2.0 mL 28% NH_3OH was added. The resulting mixture was then warmed up to room temperature. After 12 h, the reaction mixture was diluted with water and subsequently extracted with ethyl acetate (3 x 20 mL). The combined organic layer was further washed with brine, dried over Na_2SO_4, filtered and concentretd to dryness under reduced pressure. Purification by silica gel chromatography

(hexanes/ethyl acetate) followed by recrystallization from dichloromethane gave FPA0-NH_2 as a white solid in 51% yield (561.3 mg). ^{1}H NMR (400 MHz, DMSO-*d6*): δ 6.98 (s, 4H); ^{19}F NMR (377 MHz, , DMSO-*d6*): δ -162.11 – -162.00 (m, 4F), -146.21 – -146.12 (m, 4F).

Synthesis of FPA6a-NH₂

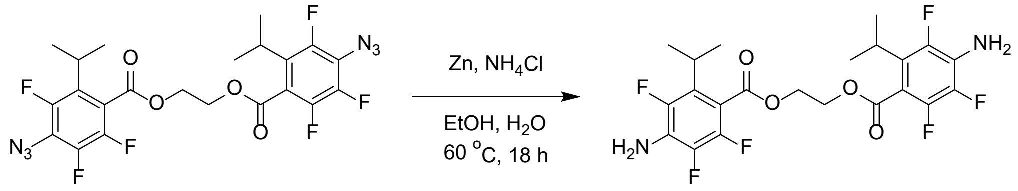

FPA6a **FPA6a-NH$_2$**

Ethane-1,2-diyl bis(4-amino-2,3,5-trifluoro-6-isopropylbenzoate) (FPA6a-NH$_2$): To a 25-mL RBF equipped with a magnetic stir bar, **FPA6a** (163.32 mg, 0.3 mmol) and NH_4Cl (160.47 mg, 3.0 mmol) were dissolved in 5.0 mL EtOH and 1.0 mL water. To the resulting solution, Zn powder (117.67 mg, 1.8 mmol) was then added. After 18 h of stirring at 60 ^{o}C, the reaction mixture was then poured into water and subsequently extracted with ethyl acetate (10 mL x 3). The combined organic layer was further washed with brine, dried over Na_2SO_4, filtered and concentrated to dryness under reduced pressure. Purification by silica gel chromatography (50% ethyl acetate in hexanes) then gave **FPA6a-NH$_2$** as a colourless viscous oil in 68% yield (100.26 mg). ^{1}H NMR (400 MHz, CD_2Cl_2): δ 1.26 (dd, J_1 = 7.0 Hz, J_1 = 1.6 Hz, 12H), 3.00 – 3.11 (m, 2H), 4.08 (brs, 4H), 4.58 (s, 4H); ^{19}F NMR (377 MHz, CD_2Cl_2): δ -159.76 – -159.67 (m, 2F), -144.96 – -144.87 (m, 2F), -138.15 (t, *J* = 10.9 Hz, 2F).

Synthesis of FPA8a

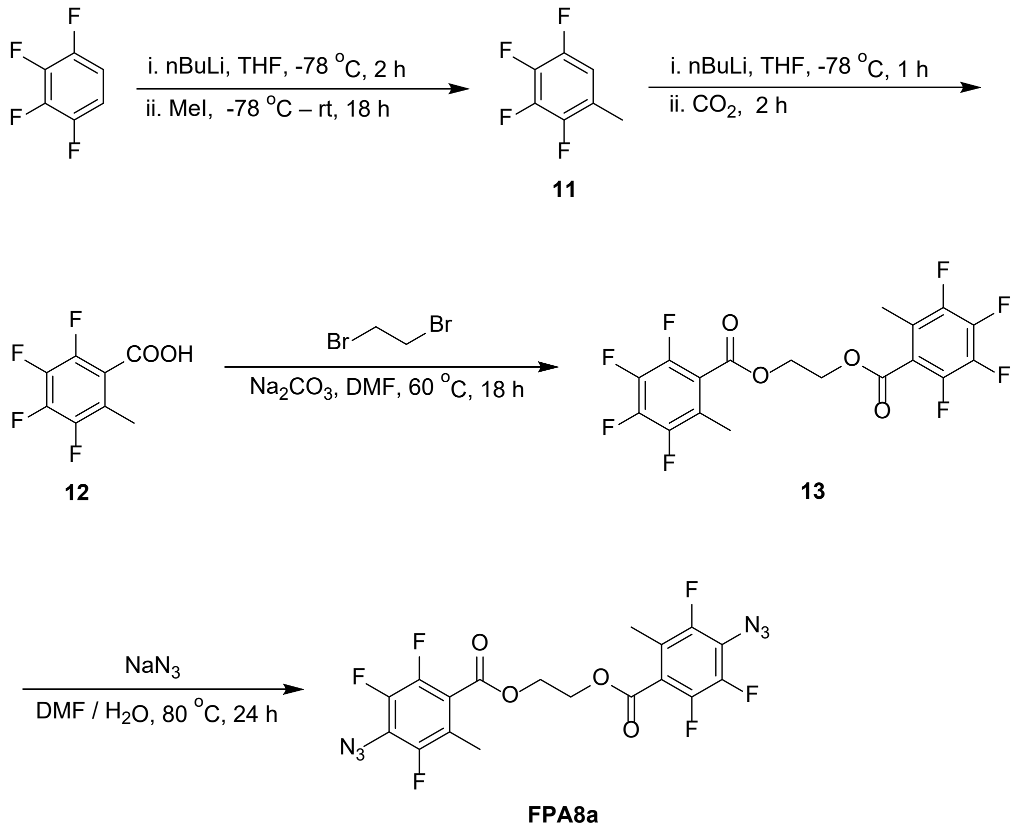

Synthesis of 1,2,3,4-tetrafluoro-5-methylbenzene (11): To an oven dried 2-neck 250-mL RBF equipped with a magnetic stir bar, 1,2,3,4-tetrafluorobenzene (6.0 mL, 57.1 mmol) was mixed in 175 mL anhydrous THF under an argon atmosphere. At -78 °C (dry ice-acetone bath), 2.0 M n-butyllithium in cyclohexane (32 mL, 64 mmol) was added dropwise. After 2 h of stirring at -78 °C, methyl iodide (18 mL, 285.5 mmol) was slowly added and the resulting mixture was then allowed to warm up to room temperature. After 18 h, the reaction mixture was diluted with water and subsequently extracted with diethyl ether (50 mL x 2). The combined organic layer was washed with water and brine, dried over Na_2SO_4 and concentrated under reduced pressure at room temperature to afford **11** as a light yellow volatile oil in 70% yield (6.497 g). The crude product was used in the next step without further purification. ^1H NMR (400 MHz, $CDCl_3$): δ 2.27 (t, *J* = 1.1 Hz, 3H), 6.75-6.82 (m, 1H); ^{19}F NMR (377 MHz, $CDCl_3$): δ -159.88 (td,

J = 20.3 Hz, *J* = 7.9 Hz, 1F), -156.85 (t, *J* = 6.8 Hz, 1F), -142.96 – -142.84 (m, 1F), -140.96 (m, 1F).

Synthesis of 2,3,4,5-tetrafluoro-6-methylbenzoic acid (12): To an oven dried 3-neck 250-mL RBF equipped with a magnetic stir bar, **11** (6.497 g, 39.59 mmol) was dissolved in 115 mL anhydrous THF under an argon atmosphere. The reaction mixture was cooled to -78 °C (dry ice-acetone bath), then 2.0 M n-butyllithium in cyclohexane (23 mL, 44.34 mmol) was added dropwise. After 1 h of stirring at -78 °C, CO_2 gas was bubbled through while allowing the reaction mixture to gradually warm up to room temperature. After 2 h, the reaction mixture was diluted with water and and the resulting aqueous layer was washed with ethyl acetate to remove unreacted organics. The aqueous layer was then acidified with 5% HCl and subsequently extracted with ethyl acetate (50 mL x 3). The combined organic layer was washed with water and brine, dried over Na_2SO_4 and concentrated to dryness under reduced pressure. The solid residue was then triturated with hexanes and filtered by suction filtration to afford **12** as off-white solid in 60% yield (4.949 g). ^{1}H NMR (400 MHz, DMSO-d_6): δ 2.31 (q, *J* = 1.3 Hz, 3H), 14.26 (s, 1H); ^{19}F NMR (377 MHz, DMSO-d_6): δ -159.01 (t, *J* = 22.3 Hz, 1F), -154.68 (td, *J* = 9.3 Hz, *J* = 2.6 Hz, 1F), -141.81 (qd, *J* = 5.6 Hz, *J* = 3.8 Hz, 1F), -140.94 – -140.84 (m, 1F).

Synthesis of ethane-1,2-diyl bis(2,3,4,5-tetrafluoro-6-methylbenzoate) (13): To an oven dried 2-neck 100-mL RBF equipped with a magnetic stir bar, **12** (4.162 g, 19.35 mmol) and Na_2CO_3 (2.385 g, 22.5 mmol) were mixed in 60 mL anhydrous DMF. After 5 min of stirring at room temperature, 1,2-dibromoethane (0.78 mL, 9.0 mmol) was slowly added and the resulting mixture was then heated to 60 °C. After 18 h, the reaction mixture was diluted with water and extracted with ethyl acetate (50 mL x 3). The combined organic layer was

subsequently washed with water and brine, dried over Na_2SO_4, filtered and concentrated to dryness under reduced pressure. Purification by silica gel chromatography (10% ethyl acetate in hexanes) afforded **13** as off-white solid in 69% yield (2.75 g). ^{1}H NMR (400 MHz, $CDCl_3$): δ 2.31 (q, *J* = 1.4 Hz, 6H), 4.68 (s, 4H); ^{19}F NMR (377 MHz, $CDCl_3$): δ -157.95 (t, *J* = 21.0 Hz, 2F), -151.59 (td, *J* = 20.5 Hz, *J* = 5.3 Hz, 2F), -140.17 – -140.07(m, 2F), -139.07 – -138.96 (m, 2F).

Synthesis of ethane-1,2-diyl bis(4-azido-2,3,5-trifluoro-6-methylbenzoate) (FPA8a): To an oven dried 2-neck 100-mL RBF equipped with a magnetic stir bar, **13** (0.796 g, 1.59 mmol) was dissolved in 50 mL anhydrous DMF. A solution of NaN_3 (0.3251 g, 5.0 mmol) in 4.5 mL water was then added and the resulting mixture was subsequently heated to 80 °C for 24 h. After cooling down to room temperature, the reaction mixture was diluted with water and extracted with ethyl acetate (50 mL x 3). The combined organic layer was washed with brine, dried over Na_2SO_4 and concentrated to dryness under reduced pressure. Purification by silica gel chromatography (10% ethyl acetate in hexanes) afforded **FPA8a** as off-white solid in 95% yield (0.9231 g). ^{1}H NMR (500 MHz, $CDCl_3$): δ 2.31 (q, *J* = 1.4 Hz, 6H), 4.66 (s, 4H); ^{19}F NMR (470 MHz, $CDCl_3$): δ -148.02 (d, *J* = 20.6 Hz, 2F), -140.18 – -140.10 (m, 2F), -129.00 – -128.94 (m, 2F).

Synthesis of FPA8b

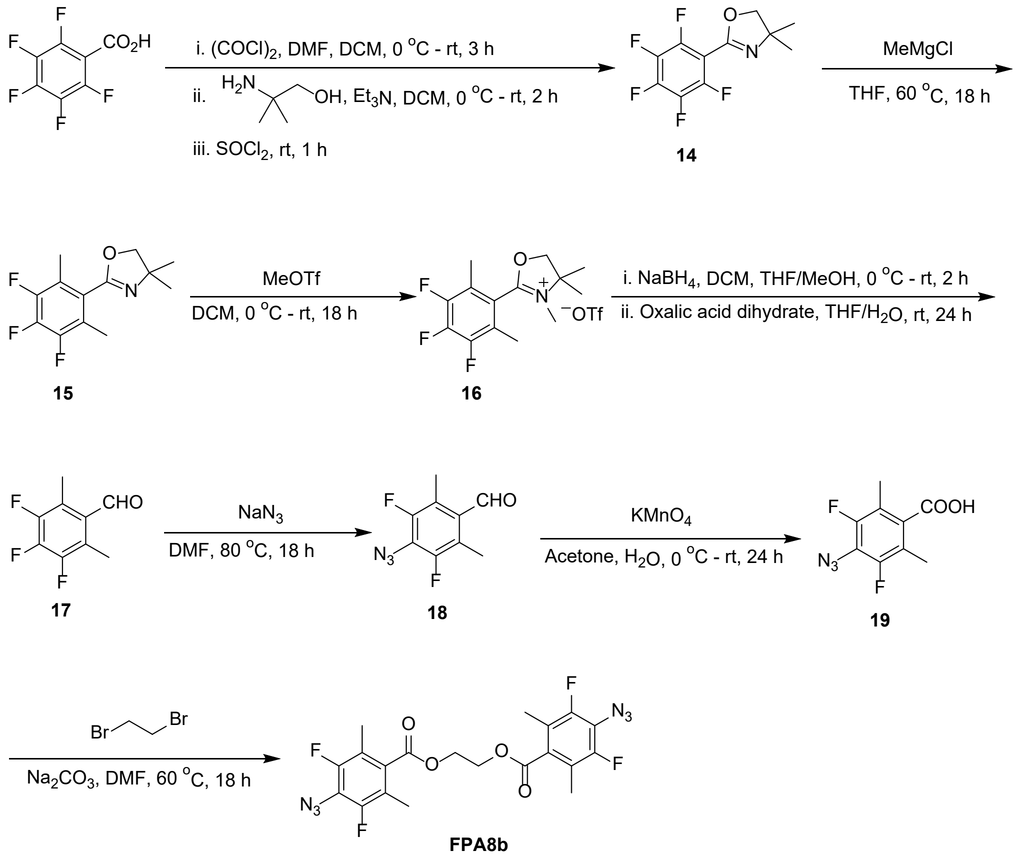

Synthesis of 4,4-dimethyl-2-(2,3,4,5,6-pentafluorophenyl)-4,5-dihydro-1,3-oxazole (14): To an oven-dried 3-neck 250-mL RBF equipped with a magnetic stir bar and 50-mL dropping funnel, 2,3,4,5,6-pentafluorobenzoic acid (10.6 g, 50 mmol) was loaded. After evacuating and purging with argon (3 cycles), 65 mL anhydrous dichloromethane and 5 drops of anhydrous DMF were added. The resulting solution was then cooled in an ice-water bath before oxalyl chloride (5.2 mL, 60 mmol) was added dropwise. After 3 h of stirring at room temperature, the reaction mixture was cooled again in an ice-water bath. Through the dropping funnel, a mixture of 2-amino-2-methyl-1-propanol (9.6 mL, 100 mmol) and Et_3N (17.4 mL, 125 mmol) was added dropwise. After 2 h of stirring at room temperature, the reaction mixture was sequentially acidified with 1.0 M HCl, washed with water and brine. The organic layer was then dried over Na_2SO_4 and concentrated to dryness under reduced pressure to afford an off-

white solid residue which was treated with 25 mL thionyl chloride. The resulting reaction mixture was stirred at room temperature for another 1 h before it was slowly added to chilled water and extracted with dichloromethane (100 mL x 3). The combined organic layer was further washed with brine, dried over Na_2SO_4 and concentrated to dryness under reduced pressure. Purification by silica gel chromatography (5% ethyl acetate in hexane) afforded **14** as a colourless oil in 67% yield (8.89 g). ^{1}H NMR (500 MHz, $CDCl_3$): δ 1.42 (s, 6H), 4.15 (s, 2H); ^{19}F NMR (470 MHz, $CDCl_3$): δ -161.06 – -160.94 (m, 2F), -150.06 (tt, J_1 = 20.9 Hz, J_2 = 3.6 Hz, 1F), -137.34 – -137.26 (m, 2F).

Synthesis of 4,4-dimethyl-2-(3,4,5-trifluoro-2,6-dimethylphenyl)-4,5-dihydrooxazole (15): To a dried 250-mL RBF equipped with a magnetic stir bar, **14** (8.89 g, 33.5 mmol) was loaded. Under an argon atmosphere, 100 mL anhydrous THF was added followed by the dropwise addition of 2.0 M MeMgCl in THF (35 mL, 70 mmol). After 18 h of stirring at 60 ^oC, the resulting reaction mixture was quenched by the slow addition of water and then extracted with diethyl ether (50 mL x 3). The combined organic layer was further washed with brine, dried over Na_2SO_4 and concentrated under reduced pressure. Purification by silica gel chromatography (5% ethyl acetate in hexane) then afforded **15** as light-yellow oil which solidified on standing to off-white solid in 98% yield (6.78 g). ^{1}H NMR (500 MHz, $CDCl_3$): δ 1.42 (s, 6H), 2.23 (t, J = 1.9 Hz, 6H), 4.11 (s, 2H); ^{19}F NMR (470 MHz, $CDCl_3$): δ -158.83 (t, J = 20.8 Hz, 1F), -140.11 (d, J = 20.8 Hz, 2F).

Synthesis of 3,4,4-trimethyl-2-(3,4,5-trifluoro-2,6-dimethylphenyl)-4,5-dihydrooxazol-3-ium trifluoromethanesulfonate (16): To a dried 100-mL RBF equipped with a magnetic stir bar, **15** (8.46 g, 32.89 mmol) was loaded. The RBF was then sealed, evacuated and purged with argon (3 cycles) before 40 mL dichloromethane was added. The resulting solution was

cooled to 0 °C (ice-water bath) followed by the dropwise addition of MeOTf (4.32 mL, 39.47 mmol). The reaction mixture was then stirred at room temperature for 18 h before purification by precipitation in diethyl ether afforded **16** as a white solid in 97% yield (13.47 g). ^{1}H NMR (500 MHz, CD_2Cl_2): δ 1.80 (s, 6H), 2.27 (t, *J* = 1.9 Hz, 6H), 3.26 (s, 3H), 5.11 (s, 2H); ^{19}F NMR (470 MHz, $CDCl_3$): δ -158.83 (t, *J* = 20.8 Hz, 1F), -140.11 (d, *J* = 20.8 Hz, 2F).

Synthesis of 3,4,5-trifluoro-2,6-dimethylbenzaldehyde (17): To a dried 150-mL 3-necked RBF equipped with a magnetic stir bar, **16** (13.06 g, 31 mmol) was loaded, dissolved in 30 mL dichloromethane. At 0 °C (ice-water bath), a solution of $NaBH_4$ (3.52 g, 93 mmol) in THF/MeOH (4:1, 80 mL) was then added dropwise using a dropping funnel. After 2 h of stirring at room temperature, the reaction mixture was quenched with saturated aqueous NH_4Cl and extracted with dichloromethane (50 mL x 3). The combined organic layer was then washed with brine, dried over Na_2SO_4 and concentrated to dryness under reduced pressure. The resulting yellow oil was redissolved in THF/H_2O (4:1, 125 mL) and 25 g oxalic acid dihydrate was subsequently added. After 24 h of stirring at room temperature, the reaction mixture was diluted with diethyl ether and sequentially washed with saturated aqueous $NaHCO_3$, water and brine. The organic layer was then dried over Na_2SO_4 and concentrated to dryness under reduced pressure. Purification by silica gel chromatography (100% hexane) then afforded **17** as a white solid in 64% yield (3.73 g). ^{1}H NMR (500 MHz, $CDCl_3$): δ 2.50 (t, *J* = 1.9 Hz, 6H), 10.48 (s, 1H); ^{19}F NMR (470 MHz, $CDCl_3$): δ -151.68 (t, *J* = 20.8 Hz, 1F), -140.01 (d, *J* = 20.8 Hz, 2F).

Synthesis of 4-azido-3,5-difluoro-2,6-dimethylbenzaldehyde (18): To a dried 100-mL 2-necked RBF equipped with a magnetic stir bar, **17** (1.88 g, 10 mmol) and NaN_3 (1.30 g, 20 mmol) were loaded. After 3-cycle of evacuation and purging with argon, 30 mL anhydrous

DMF was added and the mixture was subsequently heated to 80 °C for 18 h. After cooling to room temperature, the reaction mixture was poured into water and extracted with dichloromethane (50 mL x 3). The combined organic layer was then washed with brine, dried over Na_2SO_4 and concentrated to dryness under reduced pressure. Purification by silica gel chromatography (5% ethyl acetate in hexanes) then afforded **18** as a white solid in 91% yield (1.92 g). ^{1}H NMR (500 MHz, $CDCl_3$): δ 2.47 (t, *J* = 1.9 Hz, 6H), 10.46 (s, 1H); ^{19}F NMR (470 MHz, $CDCl_3$): δ -128.44 (s, 2F).

Synthesis of 4-azido-3,5-difluoro-2,6-dimethylbenzoic acid (19): To a dried 250-mL 2-necked RBF equipped with a magnetic stir bar, **18** (1.90 g, 9 mmol) was dissolved in acetone/water (1:1, 100 mL). At 0 °C (ice-water bath), $KMnO_4$ (1.71 g, 10.8 mmol) was then added portionwise. After 24 h of stirring at room temperature, the reaction mixture was diluted with water, washed with dichloromethane to remove unreacted organic and subsequently acidified with conc. HCl. The resulting aqueous layer was subsequently extracted with dichloromethane (100 mL x 3), washed with water and brine, dried over Na_2SO_4 and concentrated to dryness under reduced pressure to afford **19** as off-white solid in 44% yield (891 mg). ^{1}H NMR (500 MHz, DMSO-*d6*): δ 2.15 (s, 6H), 13.74 (s, 1H); ^{19}F NMR (470 MHz, DMSO-*d6*): δ -127.84 (s, 2F).

Synthesis of ethane-1,2-diyl bis(4-azido-3,5-difluoro-2,6-dimethylbenzoate) (FPA8b): To a dried 25-mL 2-necked RBF equipped with a magnetic stir bar, **19** (875 mg, 3.85 mmol) and Na_2CO_3 (464 mg, 4.375 mmol) were mixed in 12 mL anhydrous DMF. After 5 min of stirring at room temperature, 1,2-dibromoethane (0.15 mL, 1.75 mmol) was slowly added and the resulting mixture was then heated to 60 °C. After 18 h, the reaction mixture was poured into water and extracted with dichloromethane (50 mL x 3). The combined organic layer was

subsequently washed with water and brine, dried over Na_2SO_4, filtered and concentrated to dryness under reduced pressure. Purification by recrystallization from acetone afforded **FPA8b** as off-white solid in 98% yield (826 mg). ^{1}H NMR (500 MHz, $CDCl_3$): δ 2.15 (t, *J* = 1.9 Hz, 6H), 4.64 (s, 1H); ^{19}F NMR (470 MHz, $CDCl_3$): δ -127.16 – -127.15 (m, 4F).

^{1}H NMR of (1)

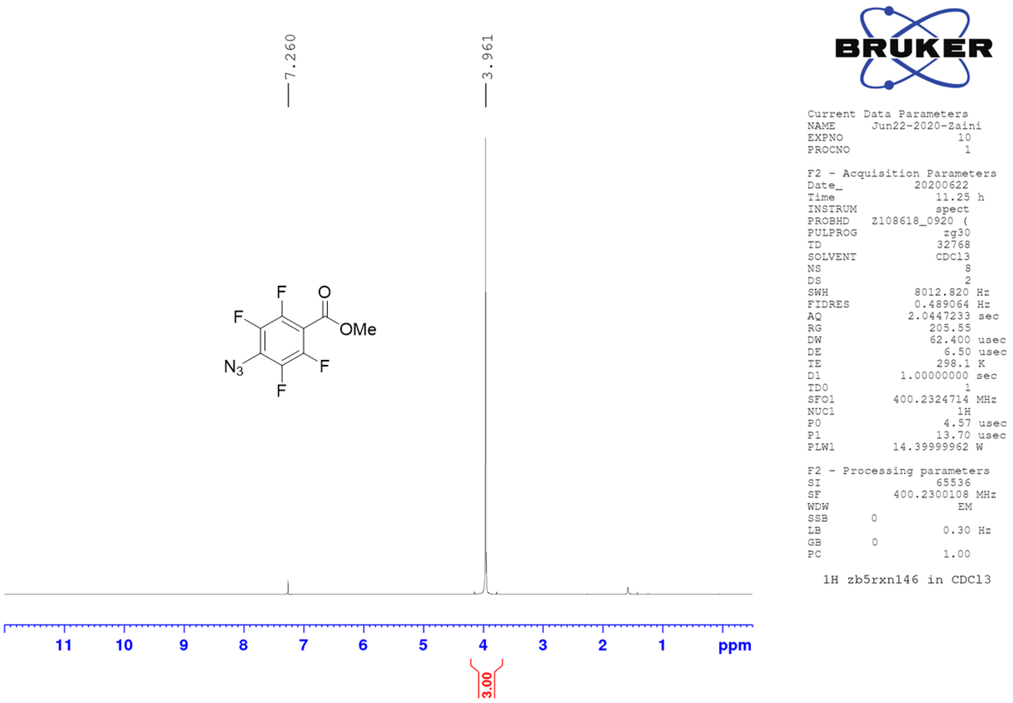

^{19}F NMR of (1)

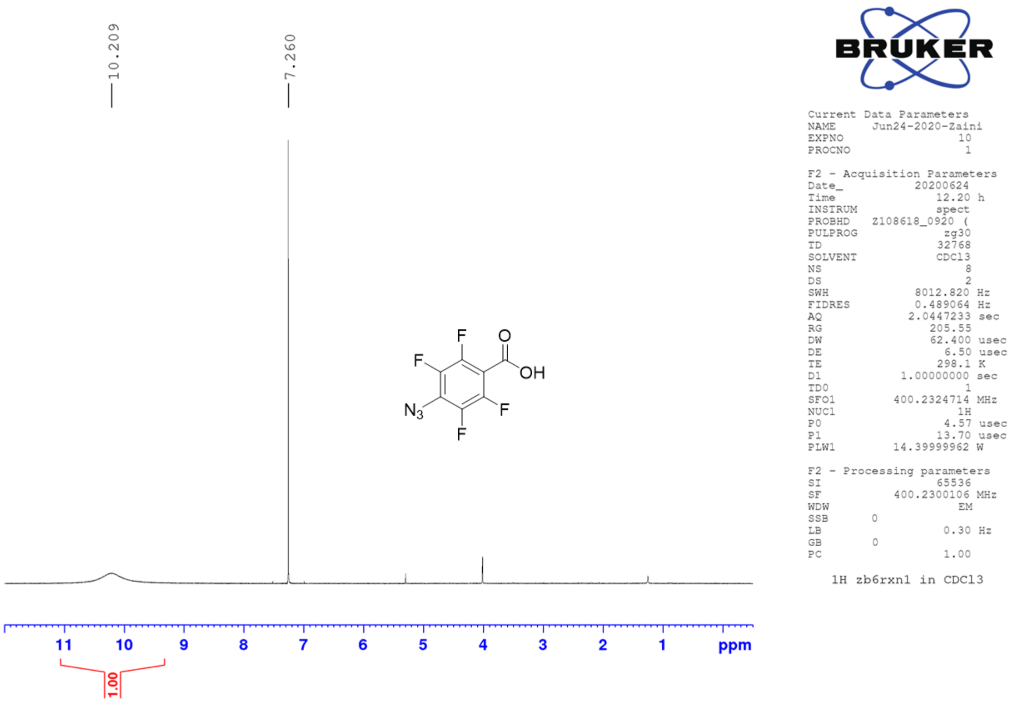

^{1}H NMR of (2)

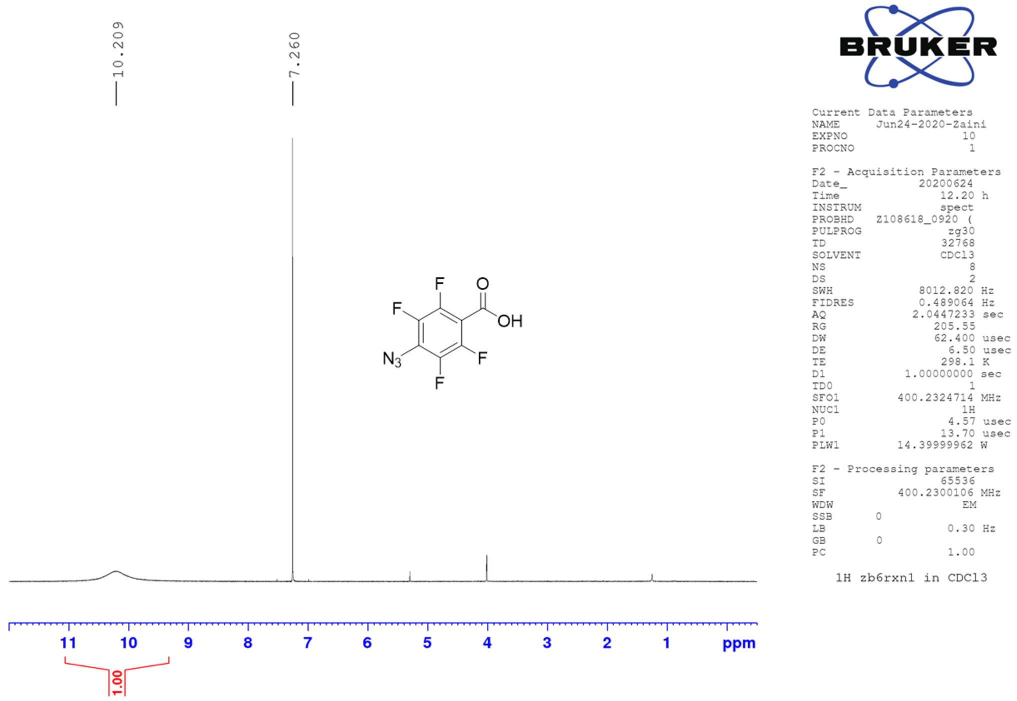

^{19}F NMR of (2)

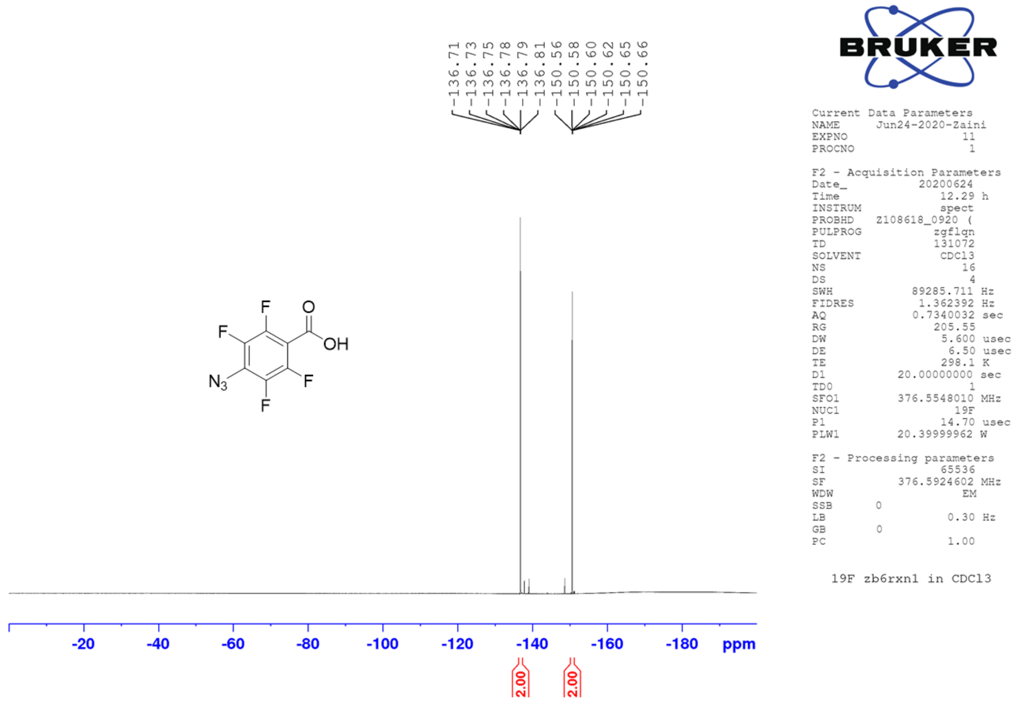

^{1}H NMR of FPA1

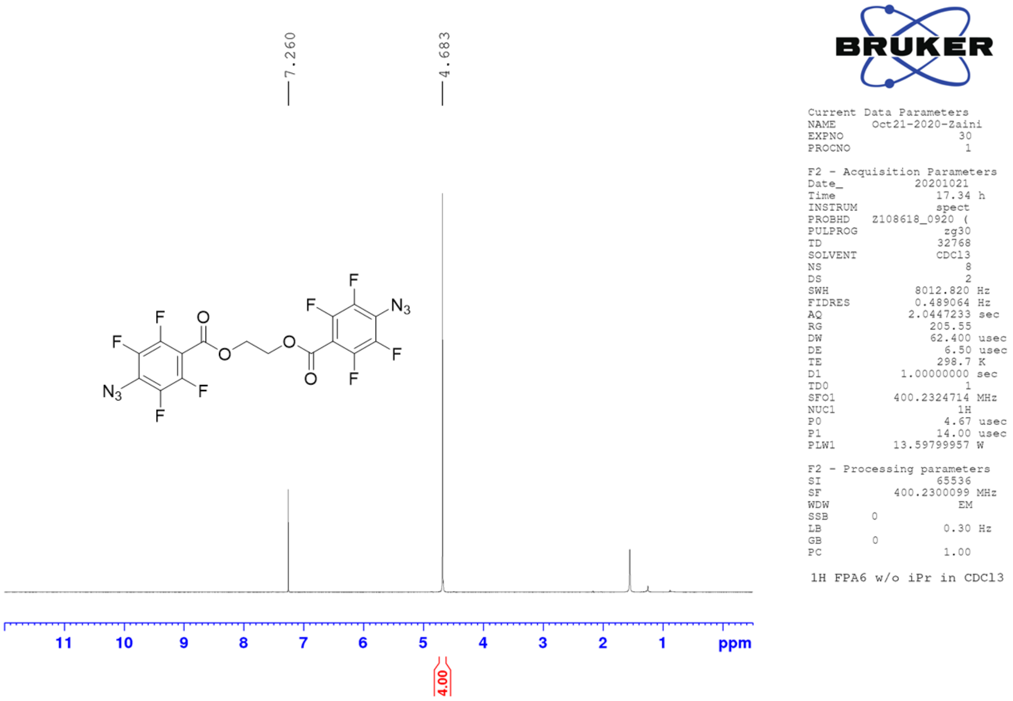

^{19}F NMR of FPA1

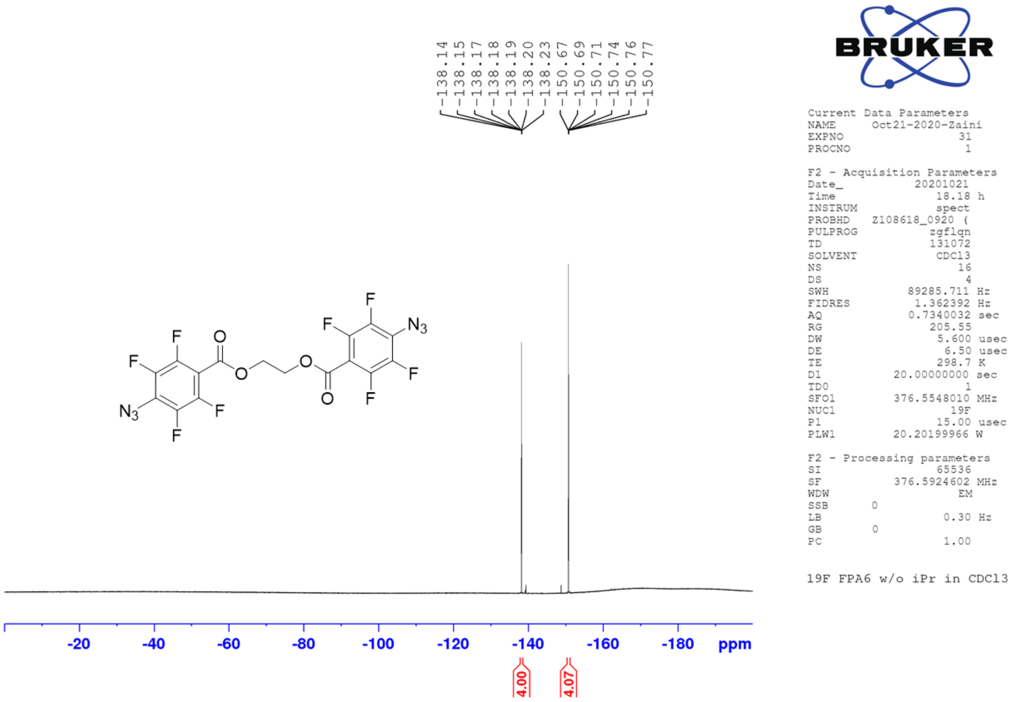

1H NMR of 2FPA1

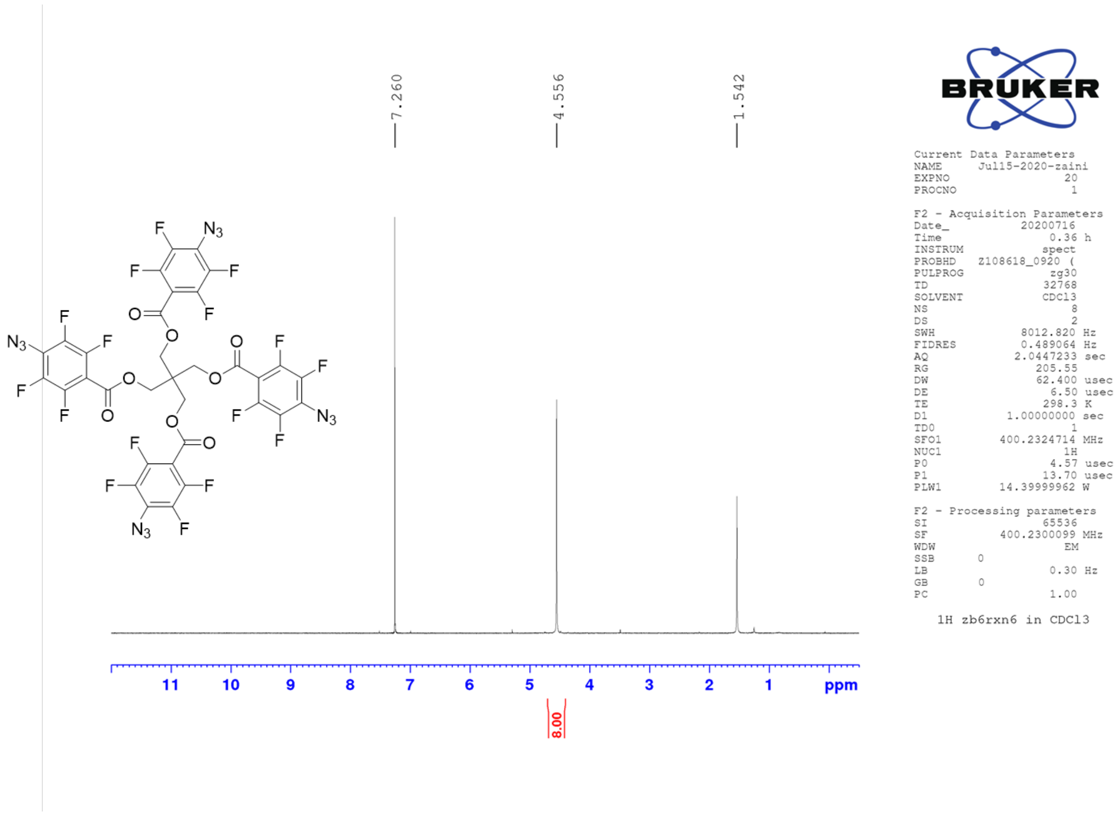

19F NMR of 2FPA1

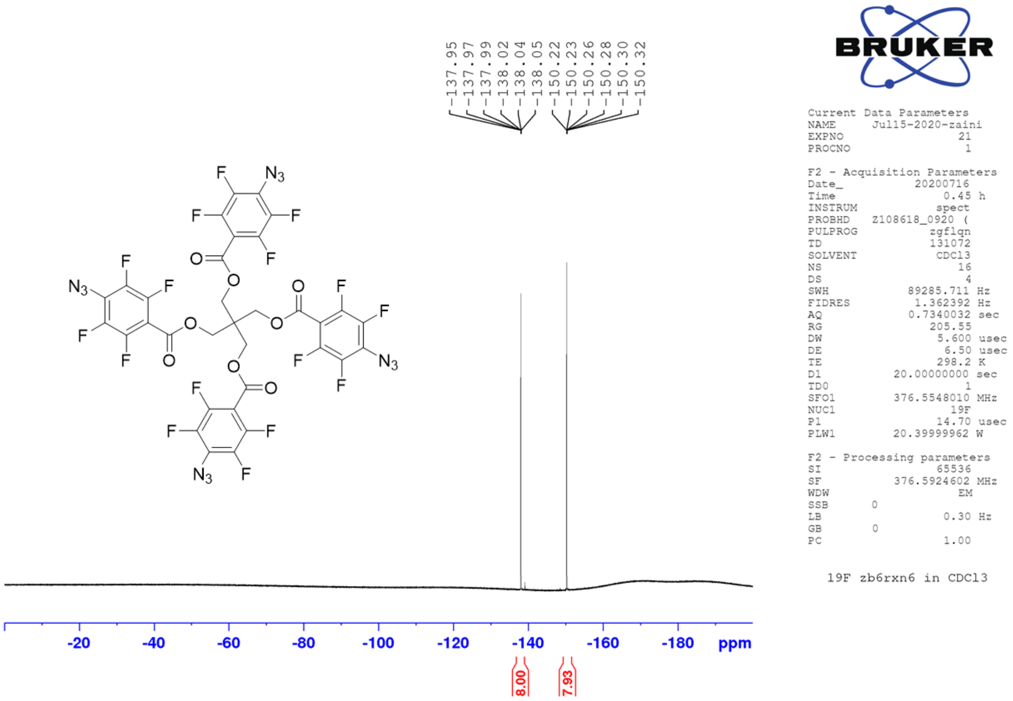

^1H NMR of (6)

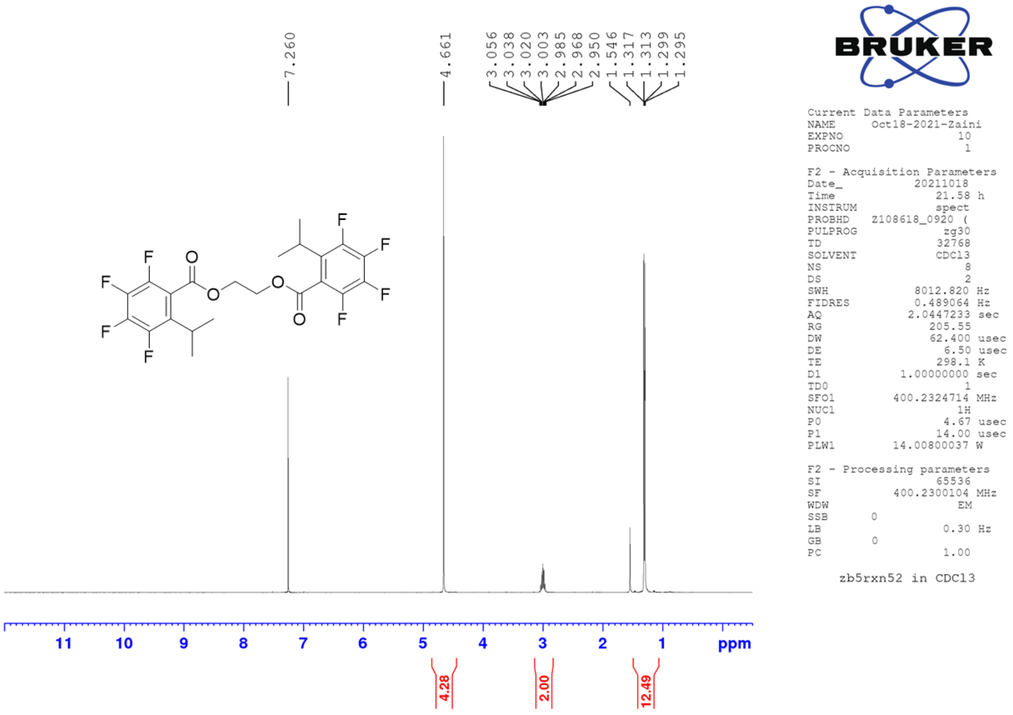

^{19}F NMR of (6)

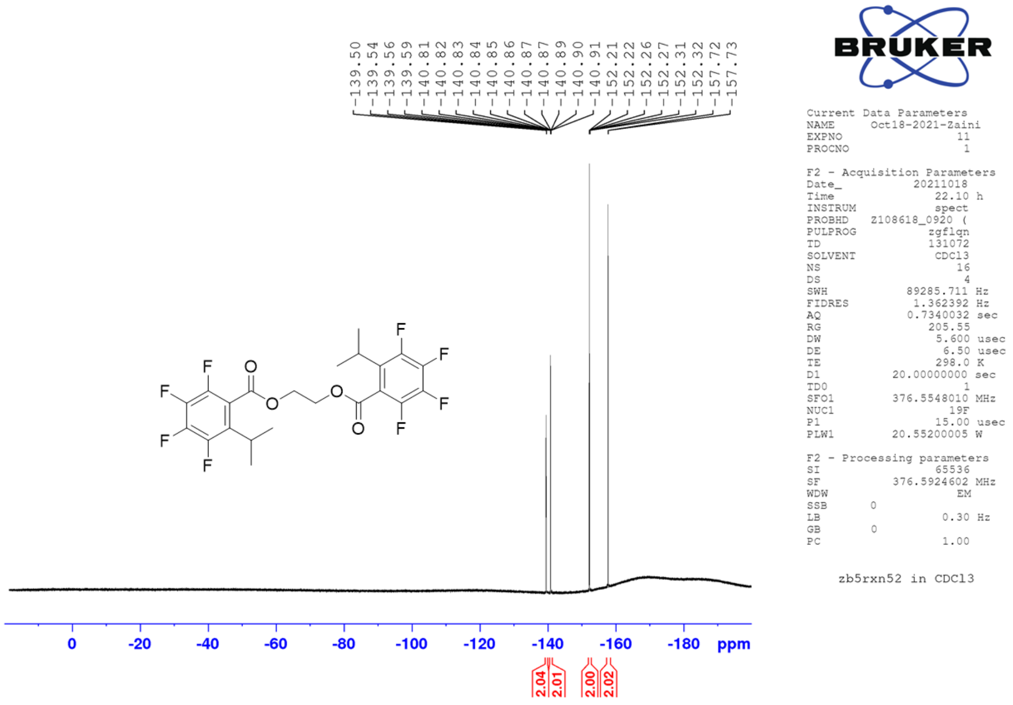

^{1}H NMR of FPA6a

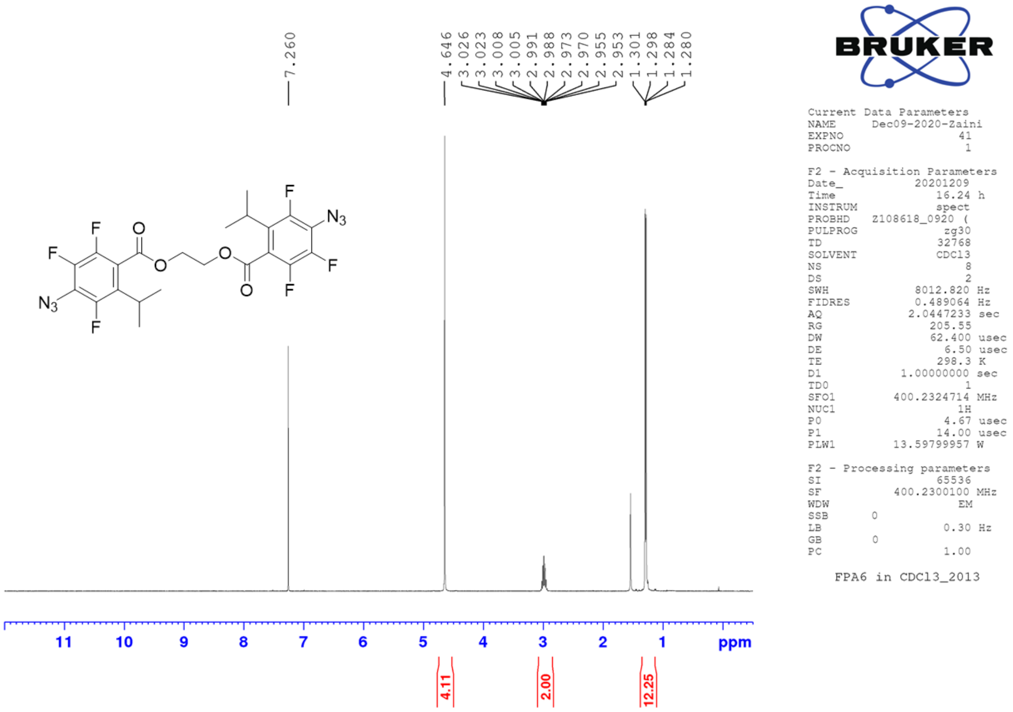

^{19}F NMR of FPA6a

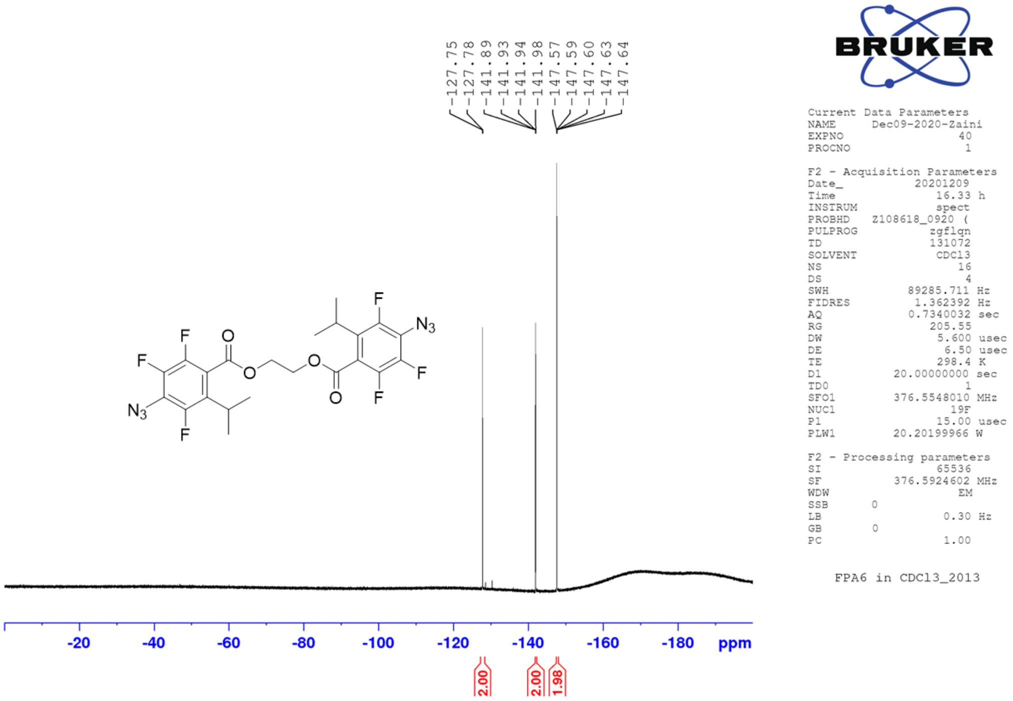

^{1}H NMR of (7)

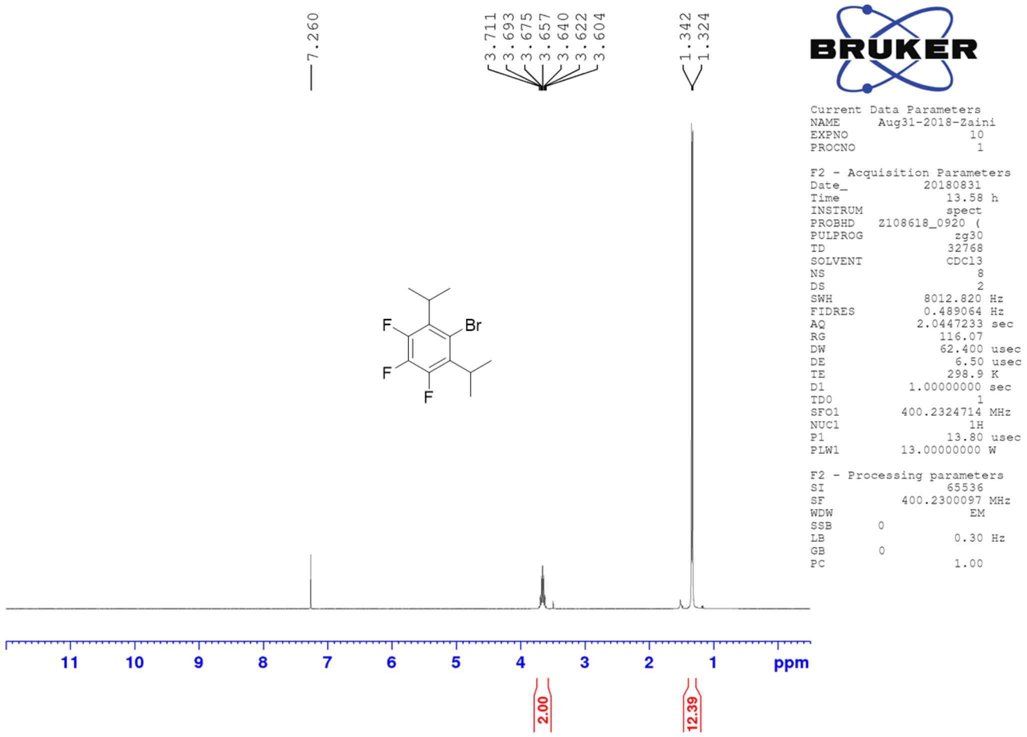

^{19}F NMR of (7)

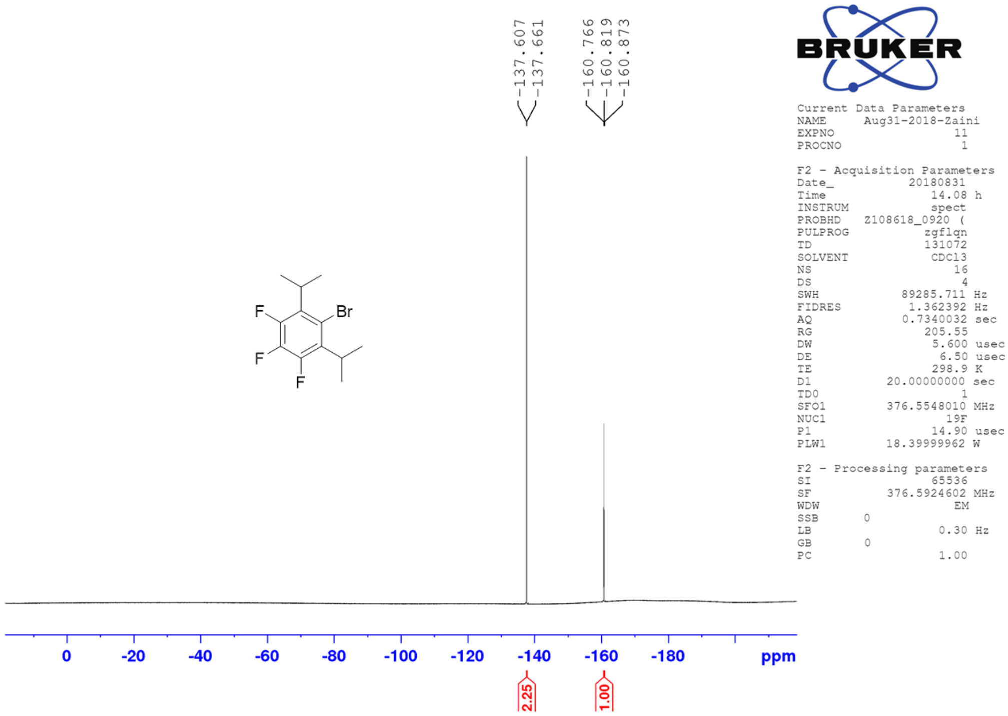

^{1}H NMR of (8)

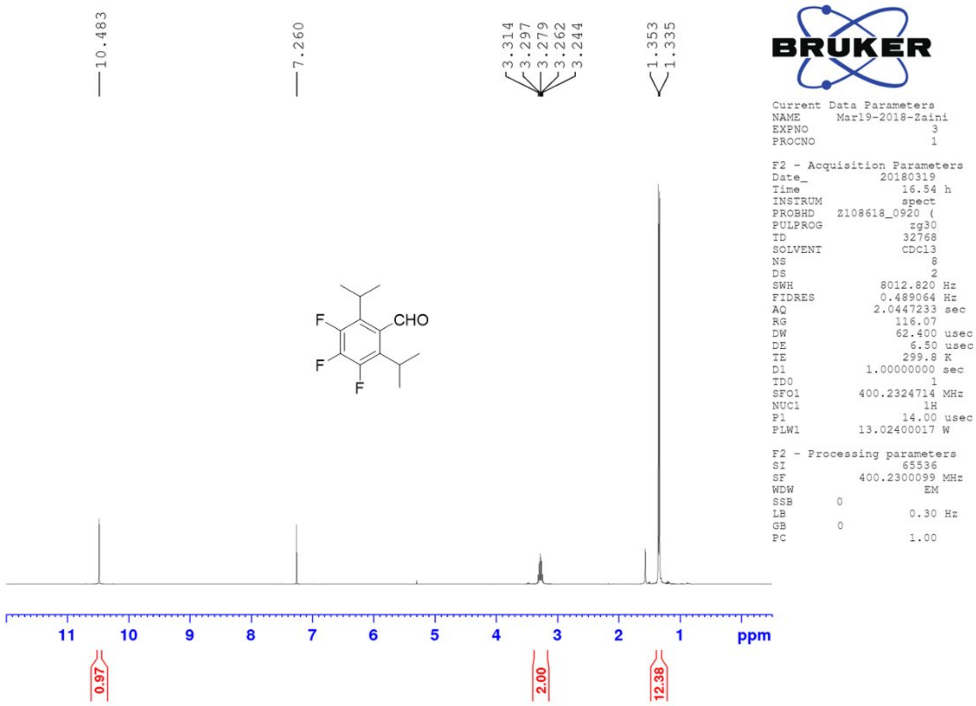

^{19}F NMR of (8)

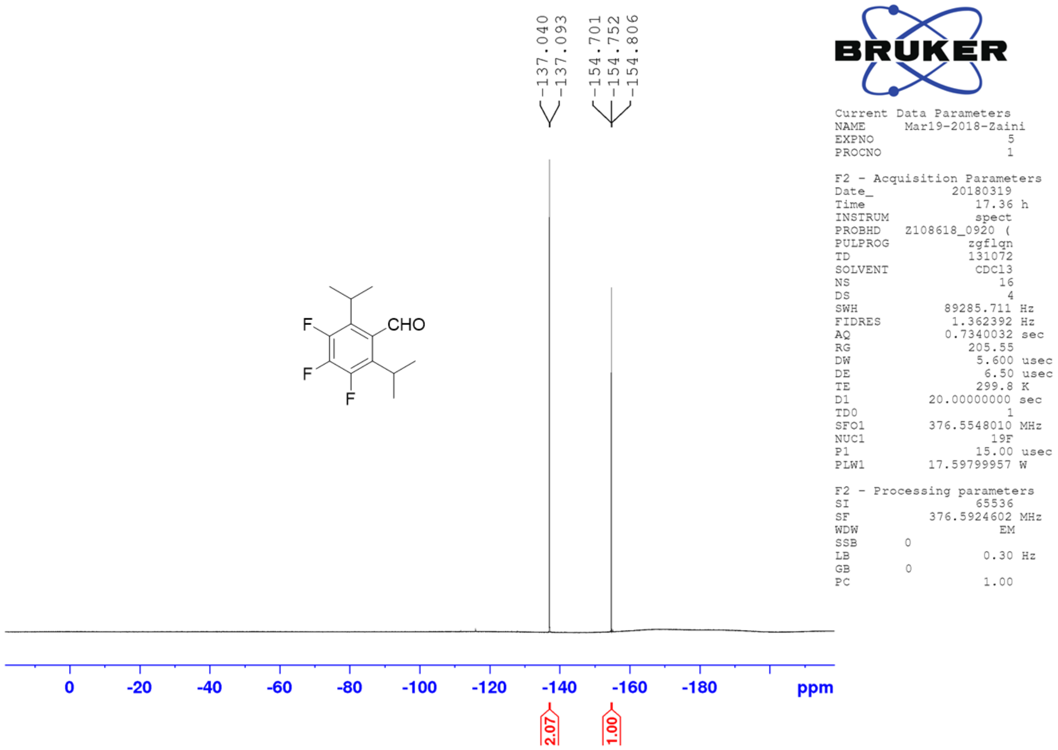

^{1}H NMR of (9)

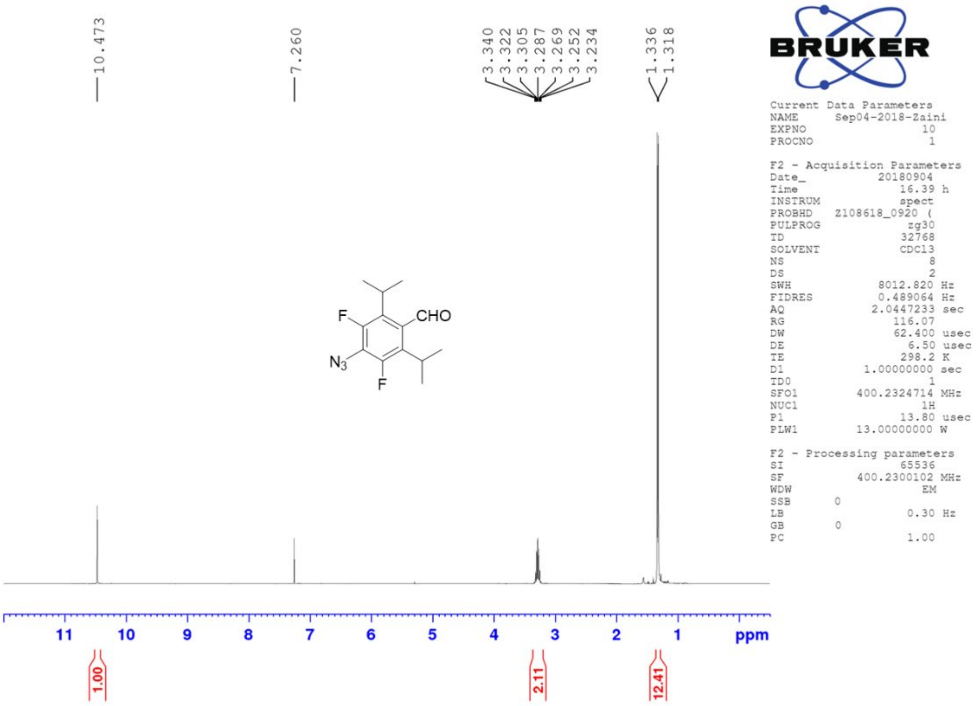

^{19}F NMR of (9)

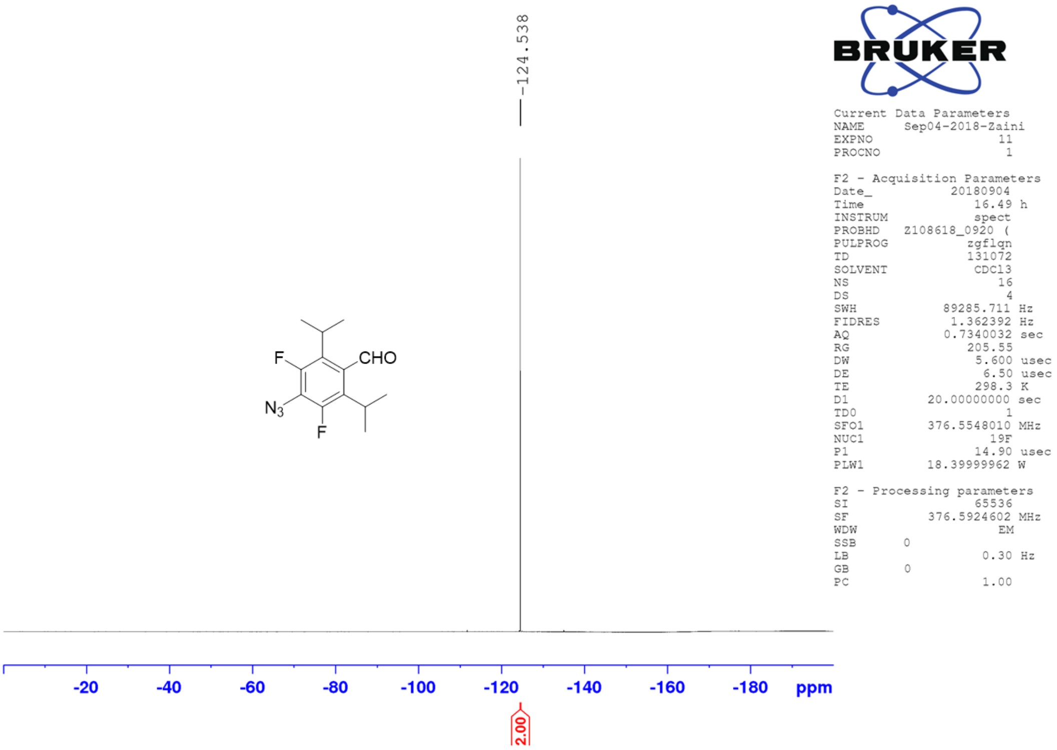

^{1}H NMR of (10)

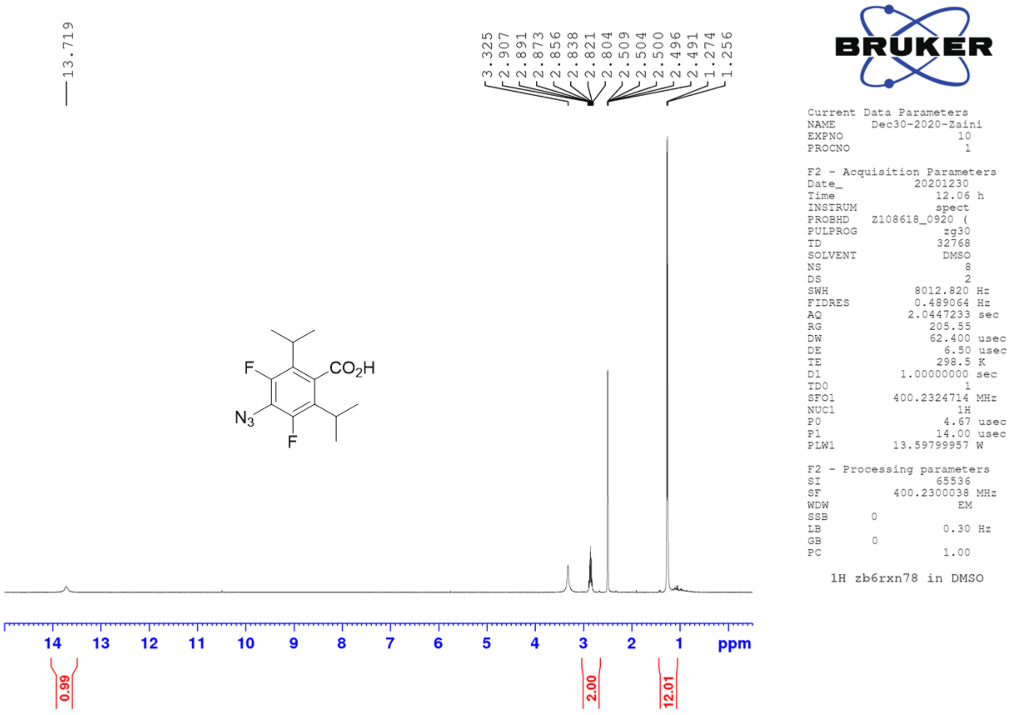

^{19}F NMR of (10)

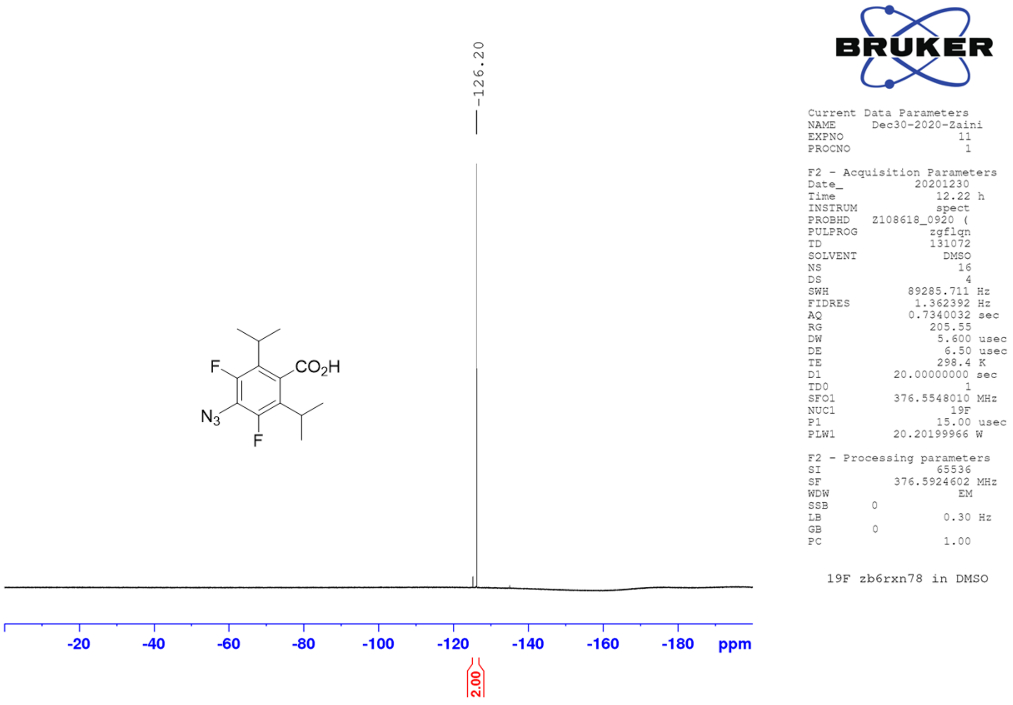

^{1}H NMR of FPA6b

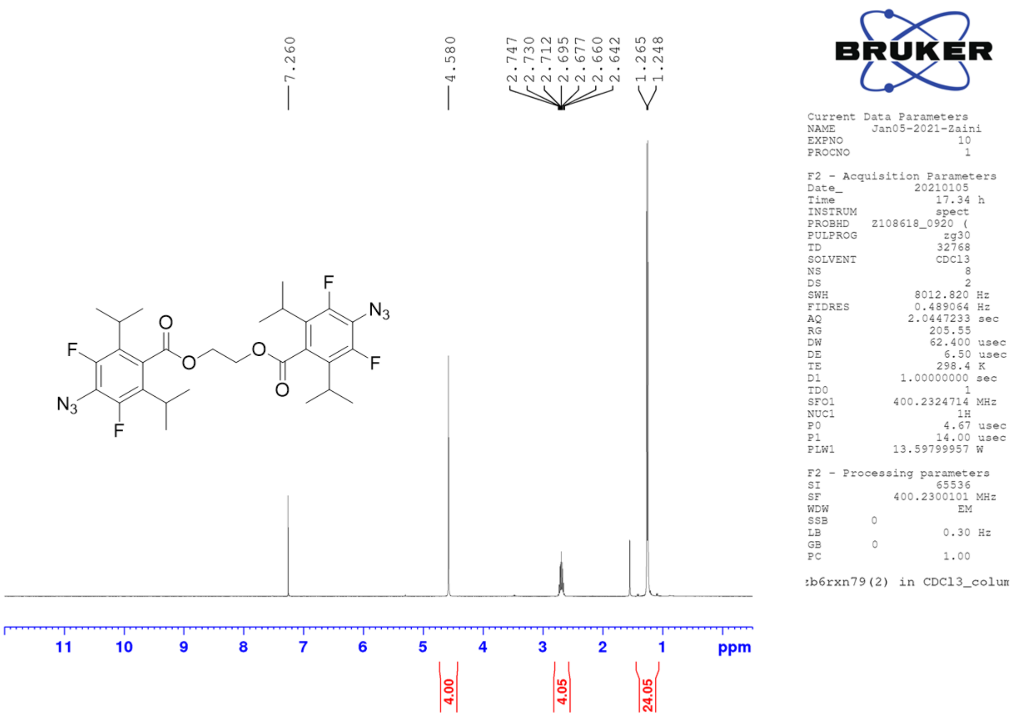

^{19}F NMR of FPA6b

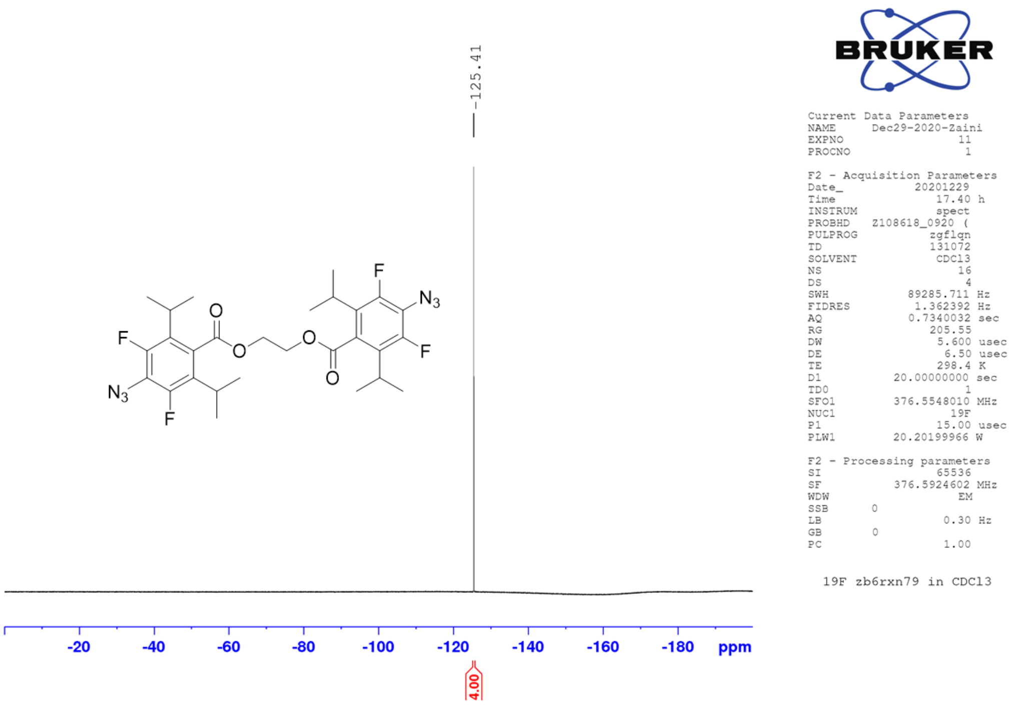

^{19}F NMR of FPA0

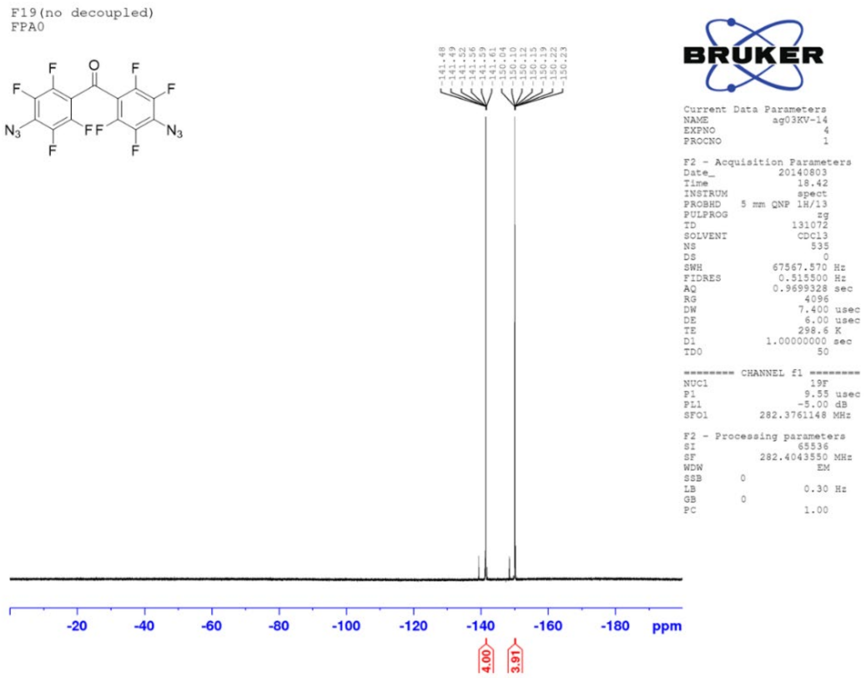

^{1}H NMR of FPA0-NH$_2$

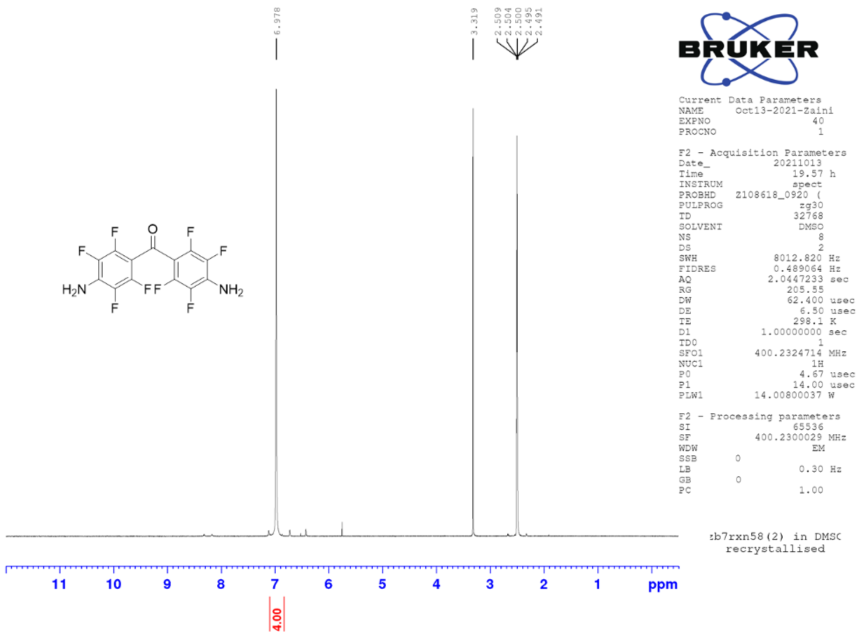

^{19}F NMR of FPA0-NH$_2$

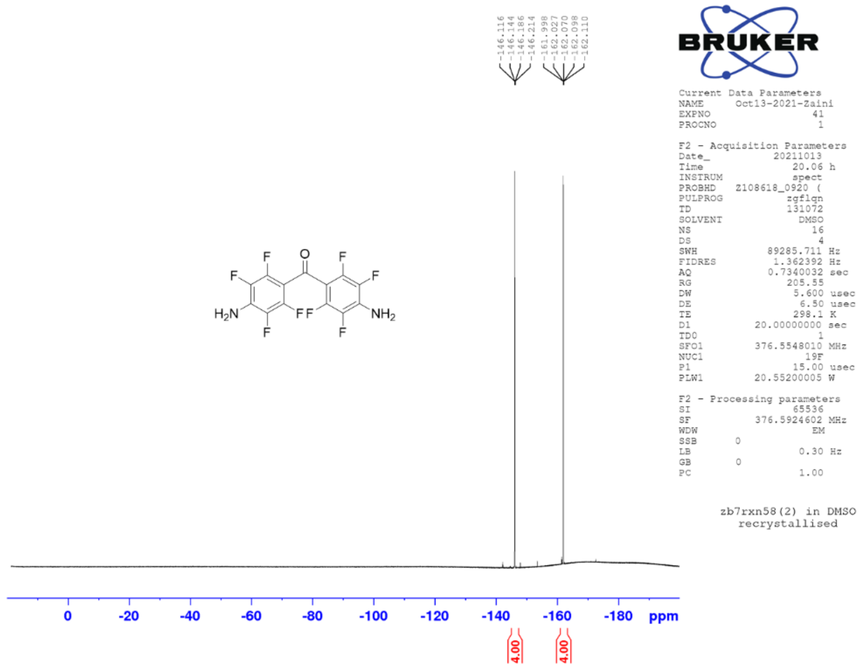

^{1}H NMR of FPA6a-NH$_2$

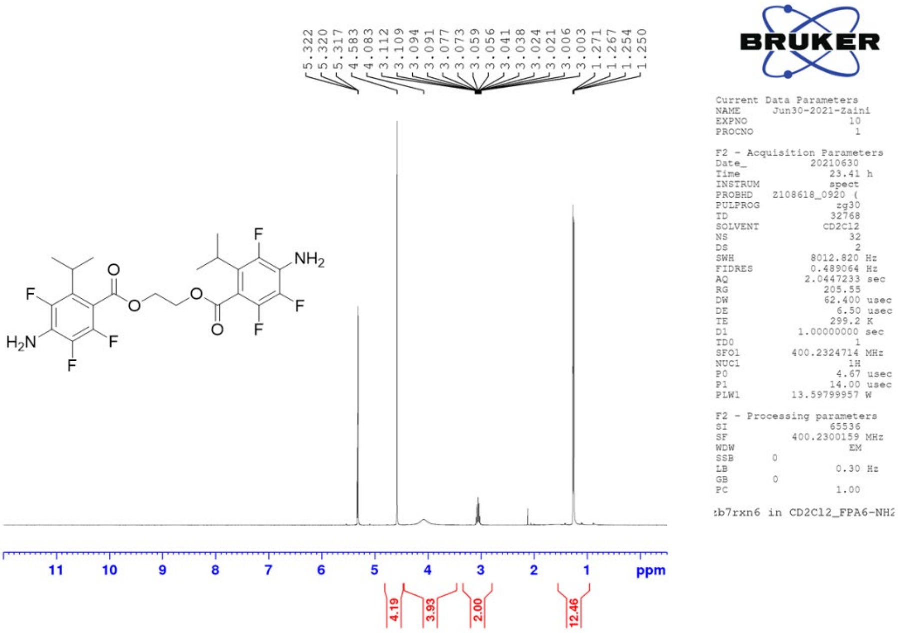

^{19}F NMR of FPA6a-NH$_2$

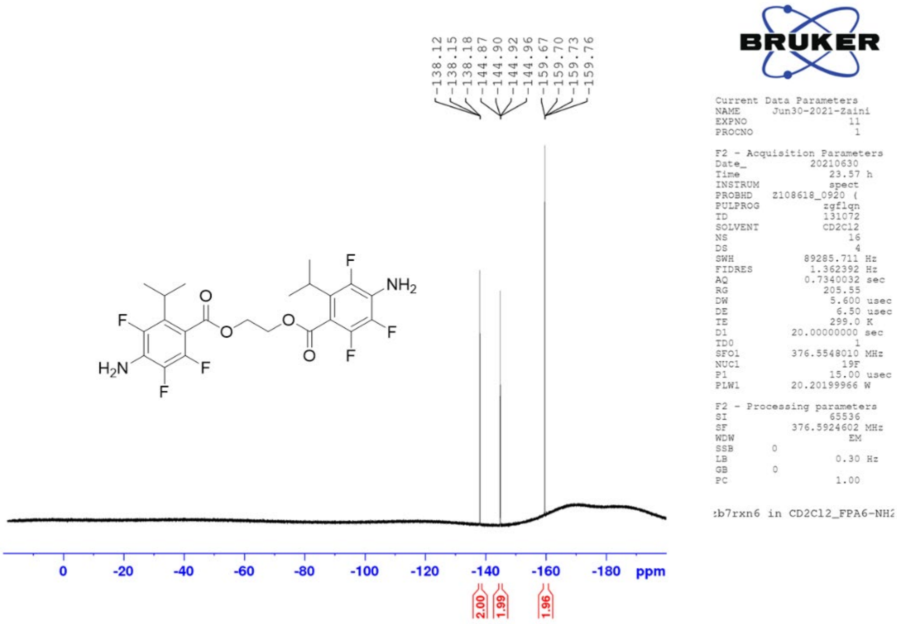

^{1}H NMR of (12)

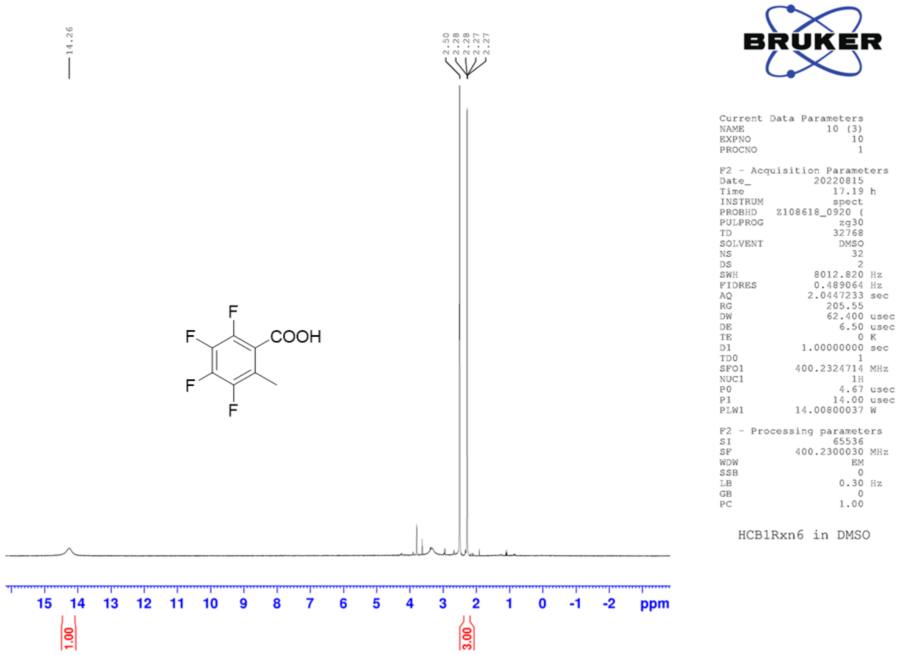

^{19}F NMR of (12)

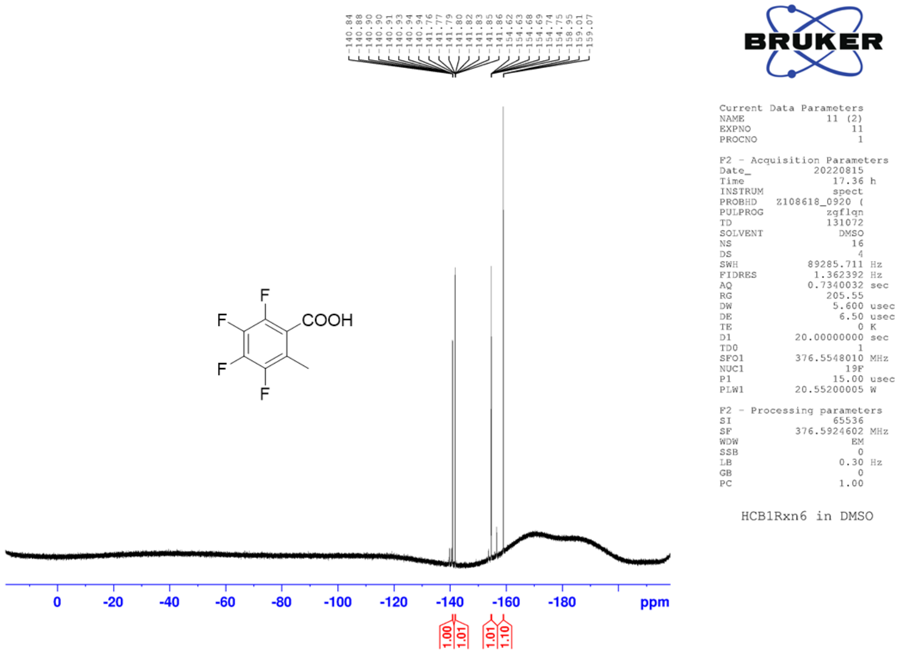

^{1}H NMR of (13)

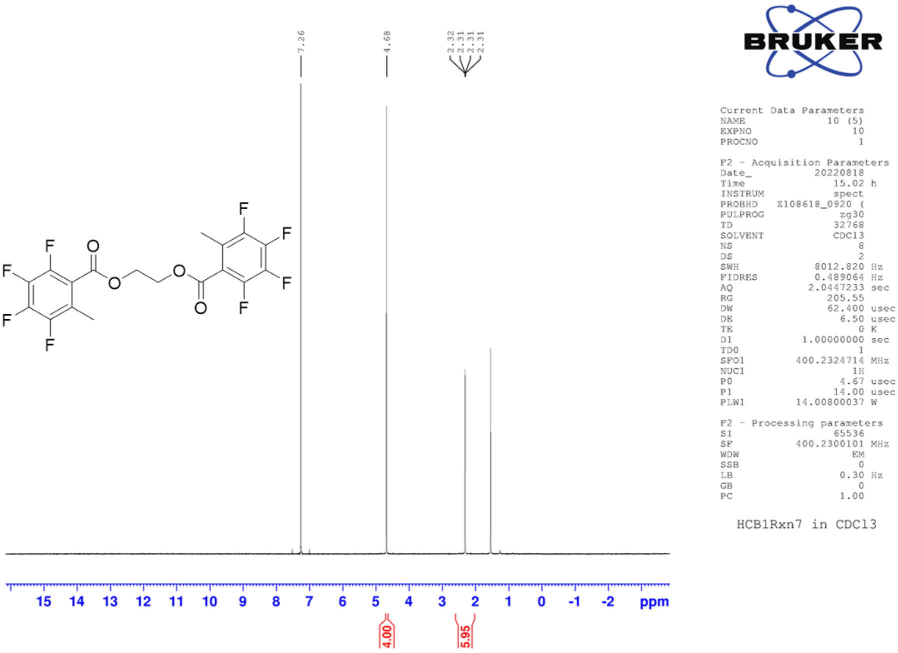

^{19}F NMR of (13)

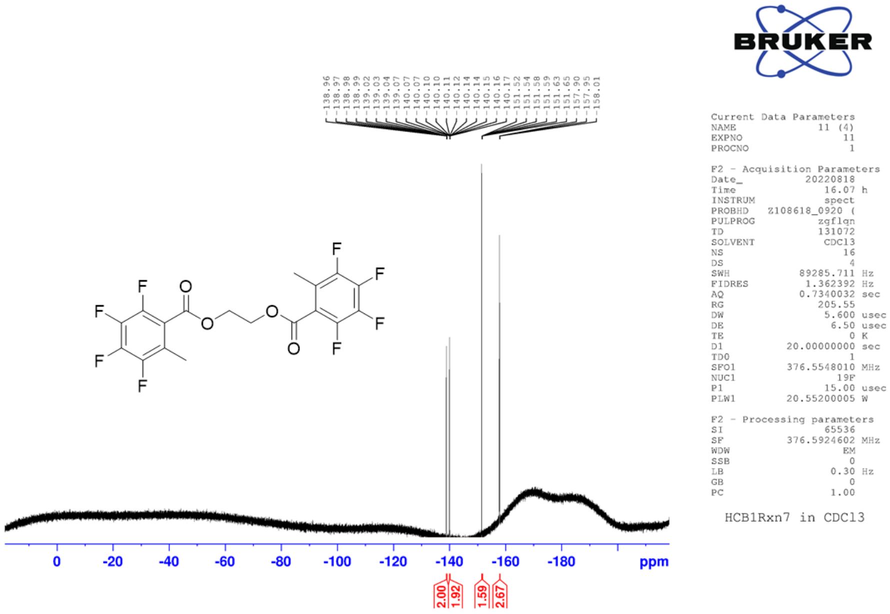

^{1}H NMR of FPA8a

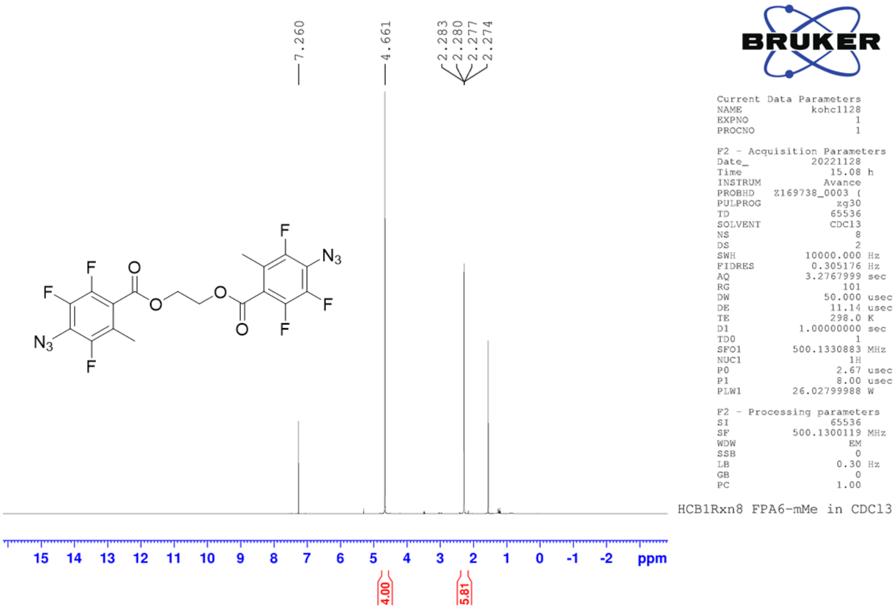

^{19}F NMR of FPA8a

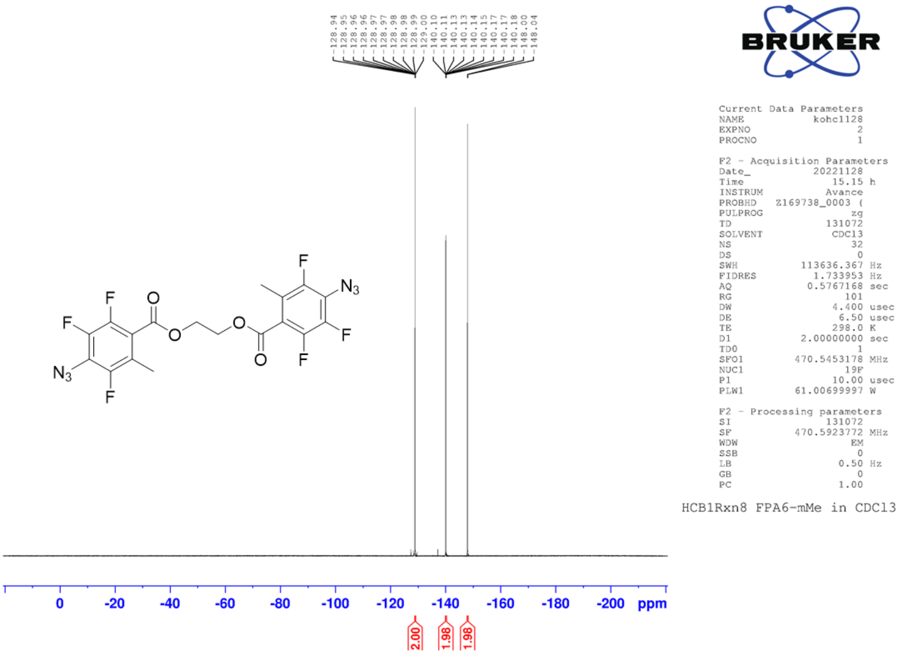

^{1}H NMR of (14)

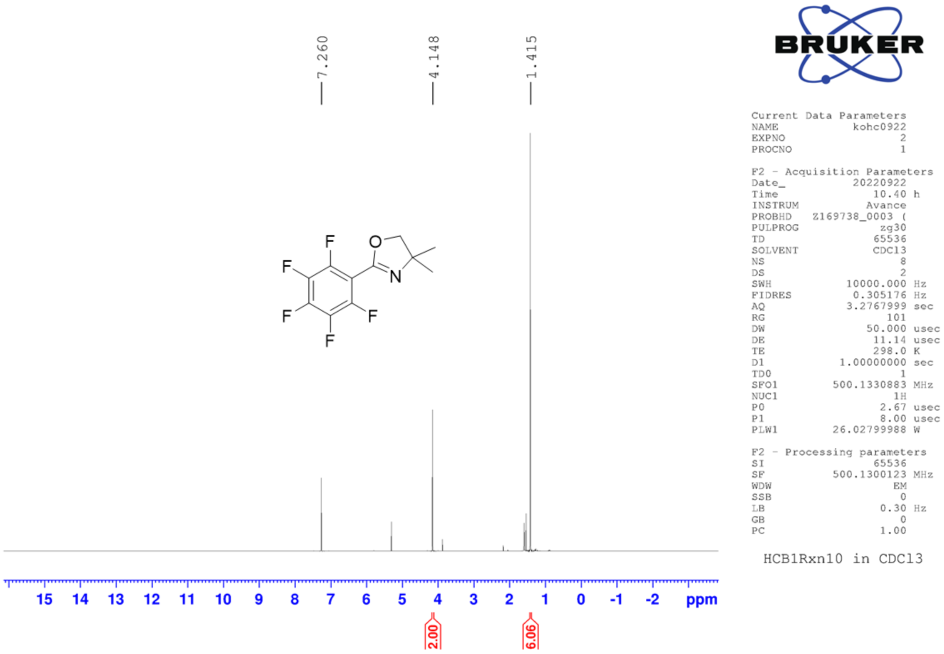

^{19}F NMR of (14)

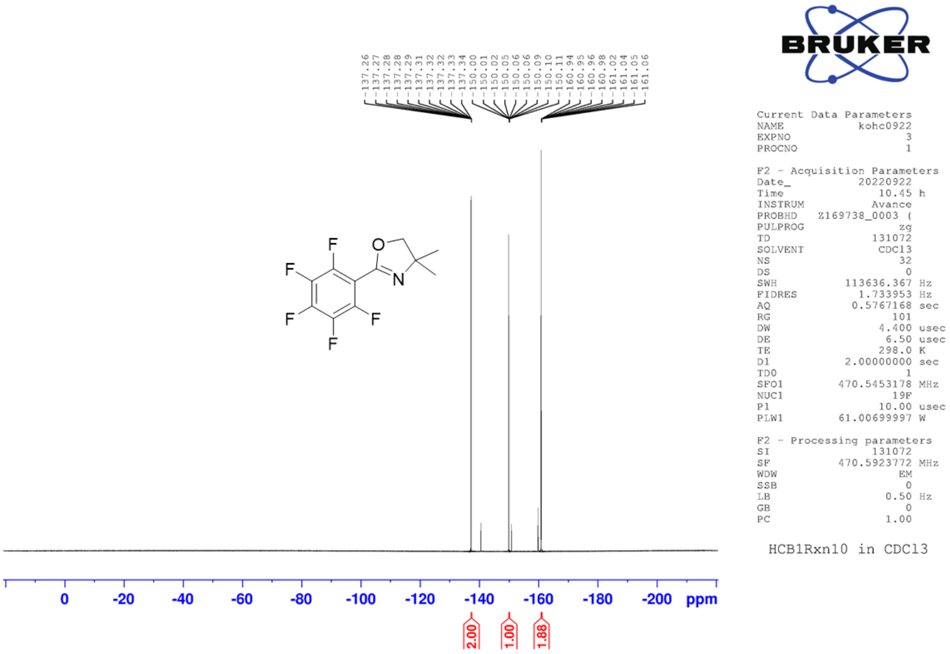

^{1}H NMR of (15)

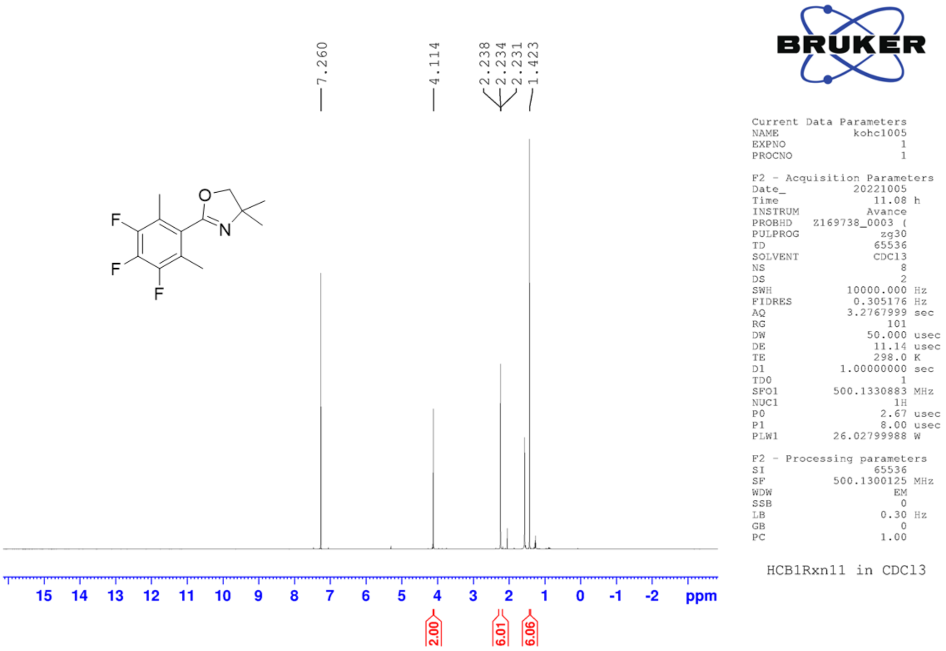

^{19}F NMR of (15)

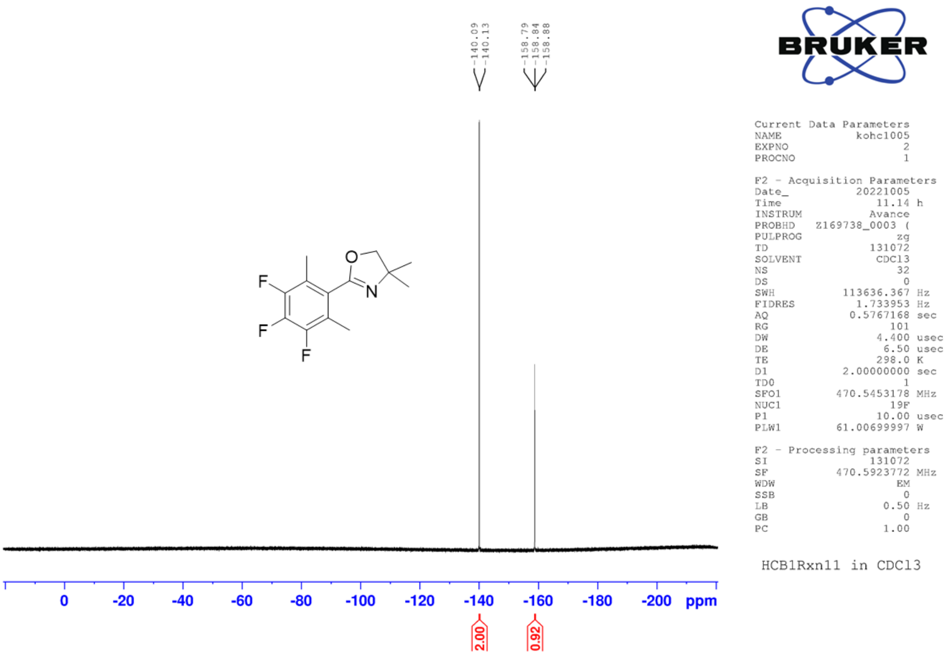

^{1}H NMR of (16)

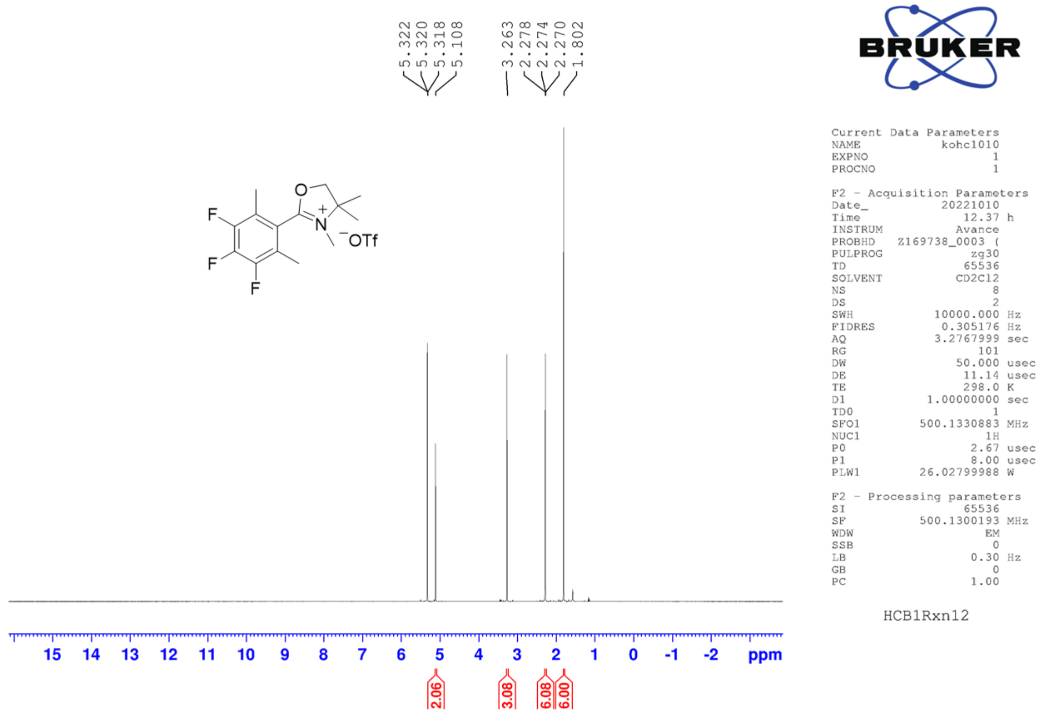

^{19}F NMR of (16)

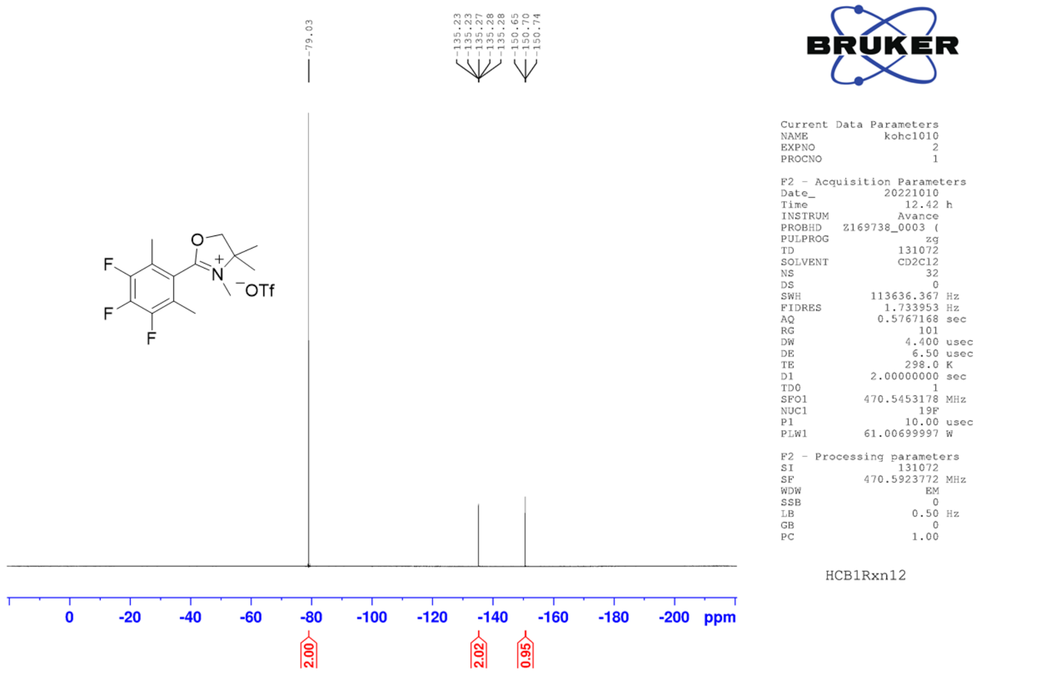

^{1}H NMR of (17)

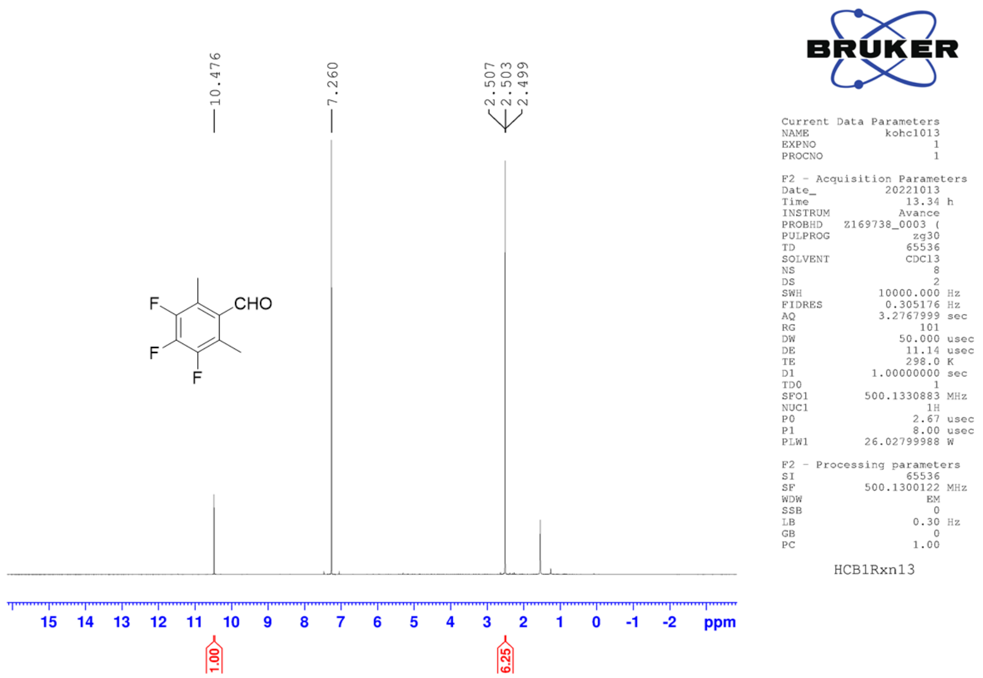

^{19}F NMR of (17)

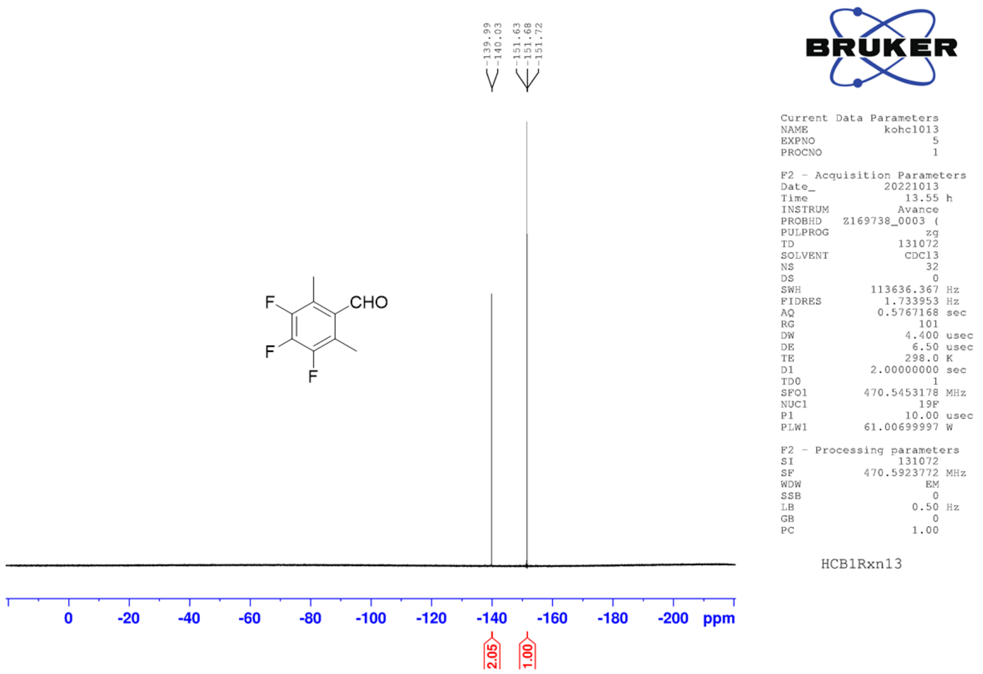

^{1}H NMR of (18)

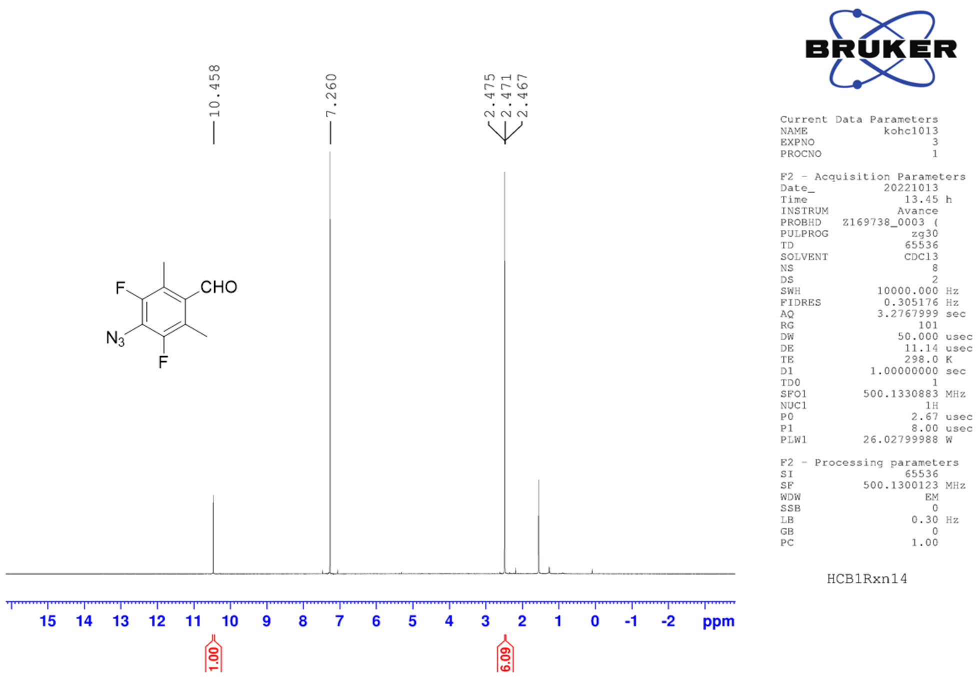

^{19}F NMR of (18)

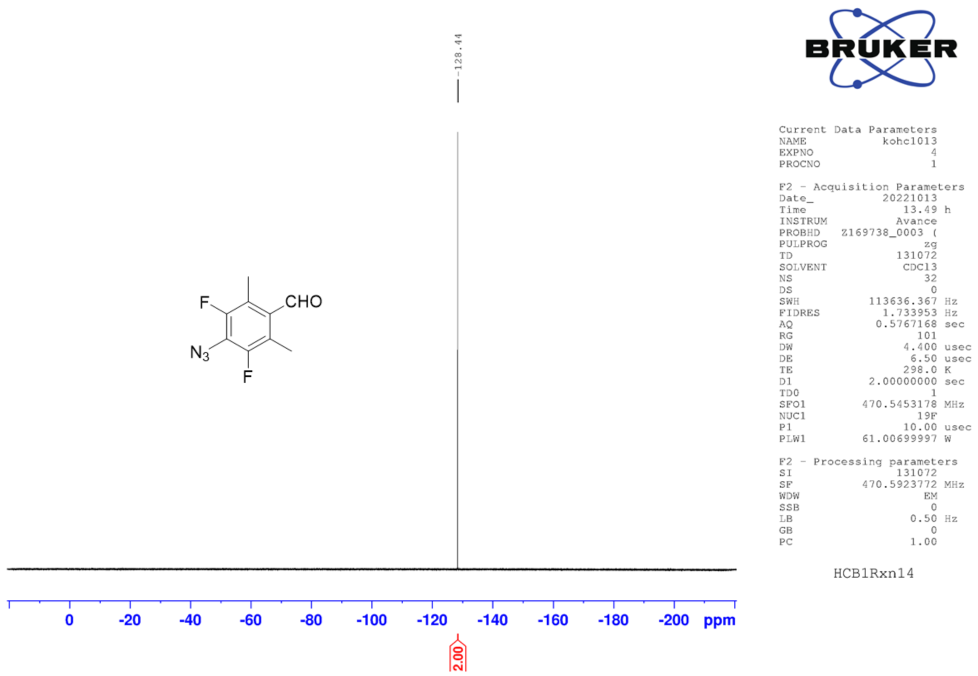

^{1}H NMR of (19)

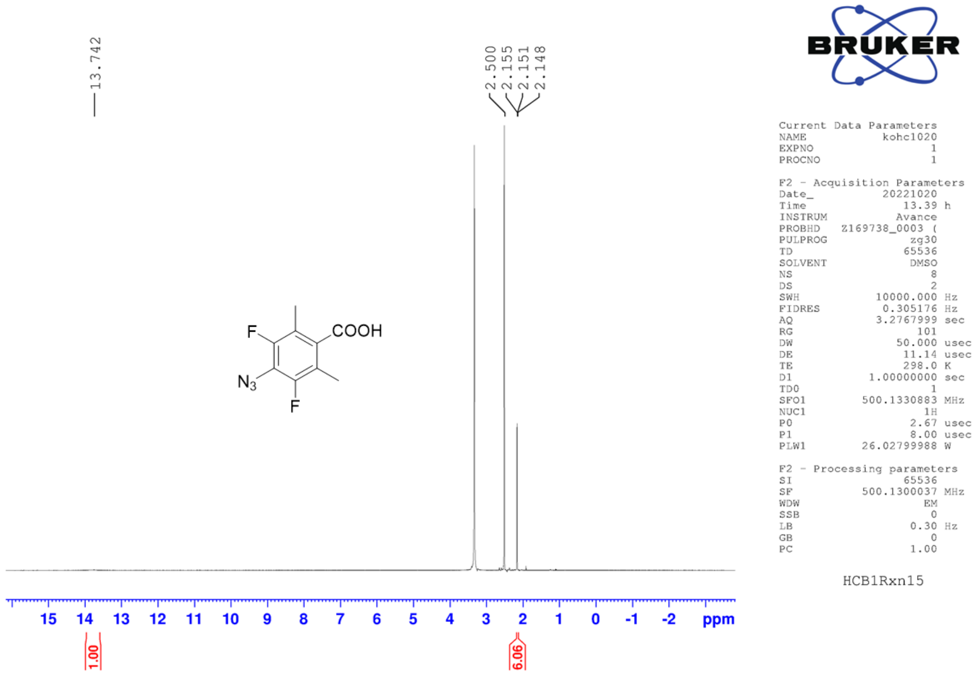

^{19}F NMR of (19)

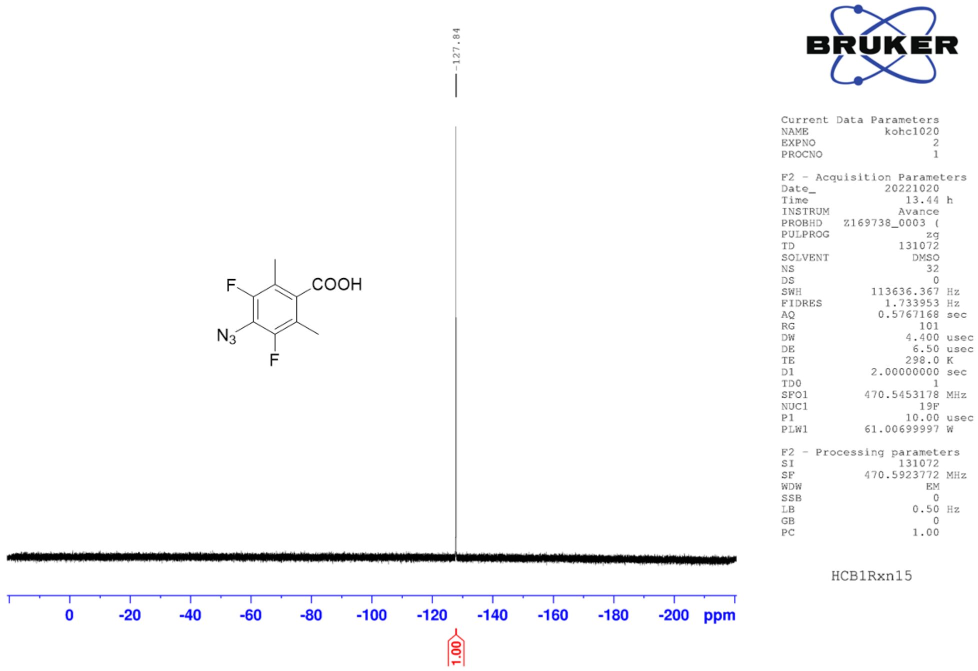

^{1}H NMR of FPA8b

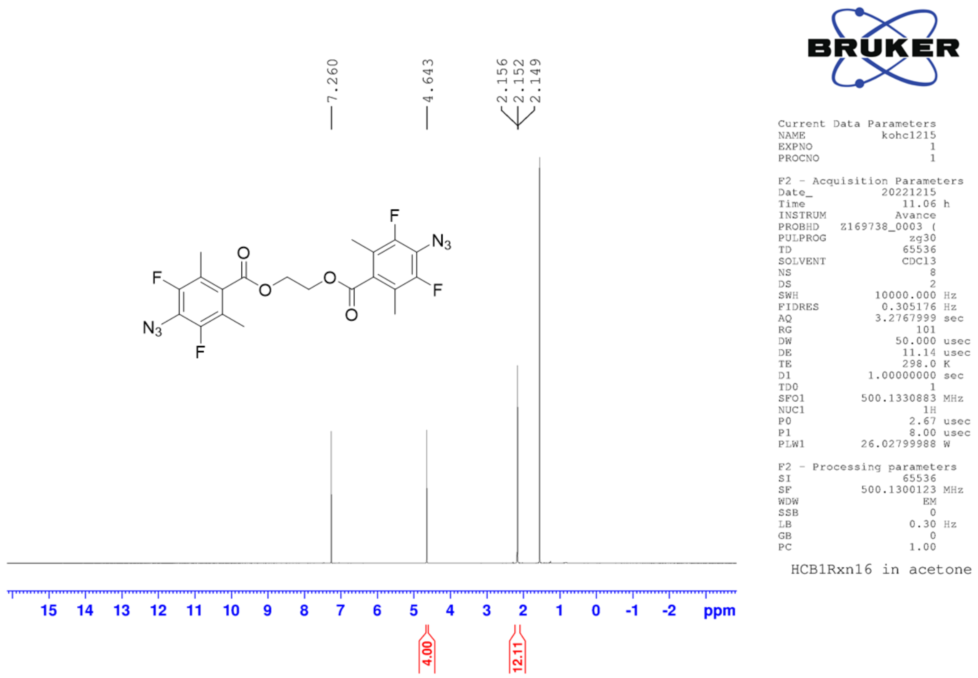

^{19}F NMR of FPA8b

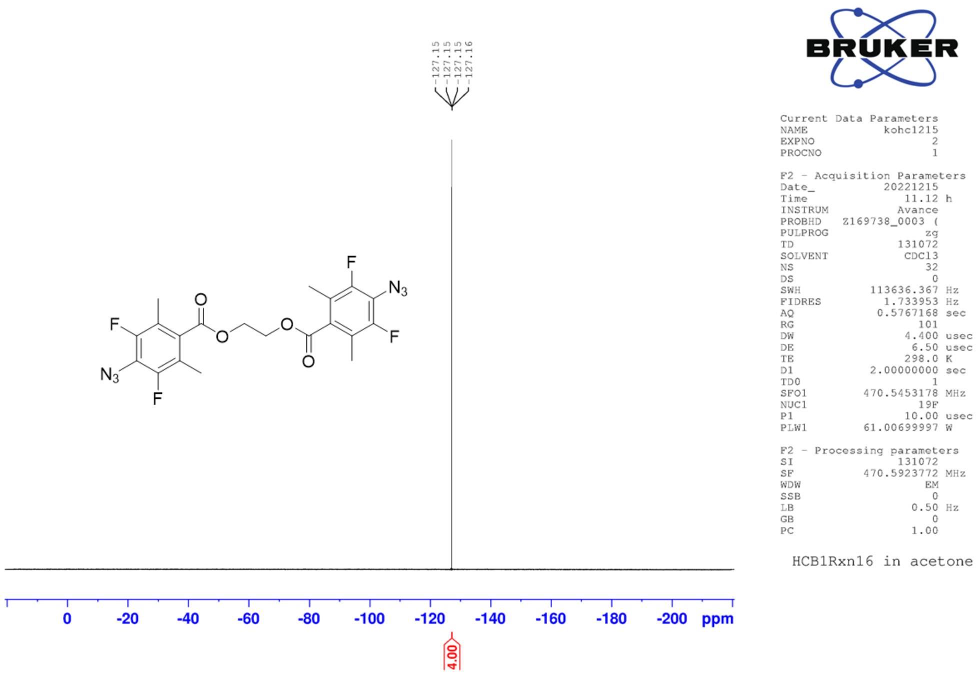

Supplement: Supplementary file 1 — Supplementary Information [file 41467_2024_50257_MOESM1_ESM.pdf]
