# Supplementary material for: Optimization of fluorinated phenyl azides as universal photocrosslinkers for semiconducting polymers
Source: Nat Commun. 2024 Jul 28;15:6354. doi: 10.1038/s41467-024-50257-5 (PMC11284223; doi:10.1038/s41467-024-50257-5)
Supplement: Supplementary file 2 — Reporting Summary [file 41467_2024_50257_MOESM2_ESM.pdf]

nature portfolio

Corresponding author(s): *Double-anonymous peer review submissions: write DAPR and your manuscript number here instead of author names.*

Solar Cells Reporting Summary

Nature Portfolio wishes to improve the reproducibility of the work that we publish. This form is intended for publication with all accepted papers reporting the characterization of photovoltaic devices and provides structure for consistency and transparency in reporting. Some list items might not apply to an individual manuscript, but all fields must be completed for clarity.

For further information on Nature Research policies, including our data availability policy, see Authors & Referees.

• Experimental design

Please check the following details are reported in the manuscript, and provide a brief description or explanation where applicable.

1. Dimensions

Area of the tested solar cells
- [x] Yes — 0.043 cm2
- [ ] No — *Explain why this information is not reported/not relevant.*

Method used to determine the device area
- [x] Yes — Device area is reported as the overlap between evaporated metal electrodes and the ITO substrates. These are confirmed by microscope examination
- [ ] No — *Explain why this information is not reported/not relevant.*

2. Current-voltage characterization

Current density-voltage (J-V) plots in both forward and backward direction
- [x] Yes — Figure 9 plots J-V curve in both forward and backward direction
- [ ] No

Voltage scan conditions
- [x] Yes — For bright curves, bias applied follows 0.0 V to +1.5 V to 0.0 V to -1.5 V to 0.0 V, where positive bias is applied on ITO electrode. Sweep takes 8 s per device, with all bright sweeps taken consecutively.
- [ ] No — *Explain why this information is not reported/not relevant.*

Test environment
- [x] Yes — Device is measured in a chamber filled with dry N2 gas.
- [ ] No — *Explain why this information is not reported/not relevant.*

Protocol for preconditioning of the device before its characterization
- [x] Yes — Two consecutive dark sweeps of 0.0 V to +3.0 V to 0.0 V to -3.0 V to 0.0 V were measured before the bright sweeps. No light soak or other pre-conditioning is applied.
- [ ] No — *Explain why this information is not reported/not relevant.*

Stability of the J-V characteristic
- [x] Yes — Repeat measurements show slow declined due to instability of these donor-acceptor system to light that are well-known in the literature. Some devices have been subjected to accelerated testing at 90 degC. The devices degraded slowly. The data is shown in Figure 9.
- [ ] No — *Explain why this information is not reported/not relevant.*

3. Hysteresis or any other unusual behaviour

Description of the unusual behaviour observed during the characterization
- [ ] Yes — *Provide a description of hysteresis or any other unusual behaviour observed during the characterization.*
- [x] No — No hysteresis observed.

Related experimental data
- [ ] Yes — *Provide a description of the related experimental data.*
- [x] No — Not applicable.

4. Efficiency

External quantum efficiency (EQE) or incident photons to current efficiency (IPCE)

☐ Yes — *Provide a description of the technique used.*
☒ No — Not used. The solar cell data are presented here only to illustrate that the crosslinking does not degrade semiconductor performance compared to reference cells fabricated and measured in the same run. The performance of the cells are very typical of what we have been making and measuring in our lab and other labs. No claim of exceptional performance is made in this paper.

A comparison between the integrated response under the standard reference spectrum and the response measure under the simulator

☐ Yes — *State where this information can be found in the text.*
☒ No — Not used. See above.

For tandem solar cells, the bias illumination and bias voltage used for each subcell

☐ Yes — *Provide a description of the measurement conditions.*
☒ No — Not applicable.

5. Calibration

Light source and reference cell or sensor used for the characterization

☒ Yes — Light source was Newport Sol2A solar simulator, calibrated with companion Newport 91150V Si solar cell.
☐ No — *Explain why this information is not reported/not relevant.*

Confirmation that the reference cell was calibrated and certified

☒ Yes — Certified by Solar Energy Research Institute Singapore (SERIS)
☐ No — *Explain why this information is not reported/not relevant.*

Calculation of spectral mismatch between the reference cell and the devices under test

☒ Yes — Mismatch factor of 1.16 was used. This accounted for 8% transmission loss at the two air-glass interfaces of chamber window and 1.08 spectral mismatch with the solar simulator, obtained from the photo-action spectrum of PBDB-T:ITIC and PM6:Y6.
☐ No — *Explain why this information is not reported/not relevant.*

6. Mask/aperture

Size of the mask/aperture used during testing

☐ Yes — *Report the size of the mask/aperture.*
☒ No — No mask/aperture applied. Whole device was illuminated.

Variation of the measured short-circuit current density with the mask/aperture area

☐ Yes — *Report the difference in the short-circuit current density values measured with the mask and aperture area.*
☒ No — Not applicable.

7. Performance certification

Identity of the independent certification laboratory that confirmed the photovoltaic performance

☐ Yes — *Identify the independent certification laboratory.*
☒ No — See response to Question 4.

A copy of any certificate(s)

☐ Yes — *Certificate copies should be provided in the Supplementary information. Please state the supplementary item number.*
☒ No — Not applicable

8. Statistics

Number of solar cells tested

☒ Yes — At least four devices of each configuration are tested, and the results are averaged. Devices of the same configuration tested are fabricated on the same substrate.
☐ No — *Explain why this information is not reported/not relevant.*

Statistical analysis of the device performance

☒ Yes — Tables in Figure 9 report the standard error of the mean.
☐ No — *Explain why this information is not reported/not relevant.*

9. Long-term stability analysis

Type of analysis, bias conditions and environmental conditions

☒ Yes — Thermal stability measurements were performed to check for the benefits of crosslinking. Substrates are placed directly on a hotplate kept at 90°C in N2 and covered with an aluminum lid to prevent illumination. Substrates are left to cool for at least 5 minutes are room temperature before measurement.
☐ No — *Explain why this information is not reported/not relevant.*
